# Supplementary material for: Predicting the risk of cancer in adults using supervised machine learning: a scoping review
Source: BMJ Open. 2021 Sep 14;11(9):e047755. doi: 10.1136/bmjopen-2020-047755 (PMC8442074; doi:10.1136/bmjopen-2020-047755)
Supplement: Author's manuscript [file bmjopen-2020-047755.draft_revisions.pdf]

BMJ Open is committed to open peer review. As part of this commitment we make the peer review history of every article we publish publicly available.

When an article is published we post the peer reviewers' comments and the authors' responses online. We also post the versions of the paper that were used during peer review. These are the versions that the peer review comments apply to.

The versions of the paper that follow are the versions that were submitted during the peer review process. They are not the versions of record or the final published versions. They should not be cited or distributed as the published version of this manuscript.

BMJ Open is an open access journal and the full, final, typeset and author-corrected version of record of the manuscript is available on our site with no access controls, subscription charges or pay-per-view fees (http://bmjopen.bmj.com).

If you have any questions on BMJ Open's open peer review process please email info.bmjopen@bmj.com

BMJ Open

# Predicting the risk of cancer in adults using supervised machine learning: a scoping review



| | |
|---|---|
| Journal: | *BMJ Open* |
| Manuscript ID | bmjopen-2020-047755 |
| Article Type: | Original research |
| Date Submitted by the Author: | 20-Dec-2020 |
| Complete List of Authors: | Alfayez, Asma; University College London; King Abdullah International Medical Research Center<br>Kunz, Holger; University College London |
| Keywords: | Information technology < BIOTECHNOLOGY & BIOINFORMATICS, Health informatics < BIOTECHNOLOGY & BIOINFORMATICS, ONCOLOGY |
| | |



SCHOLARONE™
Manuscripts

*I, the Submitting Author has the right to grant and does grant on behalf of all authors of the Work (as defined in the below author licence), an exclusive licence and/or a non-exclusive licence for contributions from authors who are: i) UK Crown employees; ii) where BMJ has agreed a CC-BY licence shall apply, and/or iii) in accordance with the terms applicable for US Federal Government officers or employees acting as part of their official duties; on a worldwide, perpetual, irrevocable, royalty-free basis to BMJ Publishing Group Ltd ("BMJ") its licensees and where the relevant Journal is co-owned by BMJ to the co-owners of the Journal, to publish the Work in this journal and any other BMJ products and to exploit all rights, as set out in our licence.*

*The Submitting Author accepts and understands that any supply made under these terms is made by BMJ to the Submitting Author unless you are acting as an employee on behalf of your employer or a postgraduate student of an affiliated institution which is paying any applicable article publishing charge ("APC") for Open Access articles. Where the Submitting Author wishes to make the Work available on an Open Access basis (and intends to pay the relevant APC), the terms of reuse of such Open Access shall be governed by a Creative Commons licence – details of these licences and which Creative Commons licence will apply to this Work are set out in our licence referred to above.*

*Other than as permitted in any relevant BMJ Author's Self Archiving Policies, I confirm this Work has not been accepted for publication elsewhere, is not being considered for publication elsewhere and does not duplicate material already published. I confirm all authors consent to publication of this Work and authorise the granting of this licence.*

For peer review only

**Predicting the risk of cancer in adults using supervised machine learning: a scoping review**

Asma Abdullah Alfayez[1,2,3], Holger Kunz[1]

[1]Institute of Health Informatics, University College London, London, UK
[2]King Abdullah International Medical Research Center, Riyadh, Saudi Arabia
[3]King Saud bin Abdulaziz University for Health Sciences, Riyadh, Saudi Arabia
***Corresponding author:** Asma Abdullah Alfayez (asma.alfayez.17@ucl.ac.uk)

**Word count:** 4375

**Keywords:** Machine learning; cancer; prediction; scoping review; artificial intelligence

**Competing interests:** None

For peer review only

## ABSTRACT

**Objectives:** The purpose of this scoping review is to identify existing supervised machine learning (ML) approaches on the prediction of cancer in asymptomatic adults, to compare the performance of ML models with each other, and to identify potential gaps in research.

**Design:** Scoping review using the population, concept, and context approach.

**Search strategy:** The literature was searched according to the following inclusion criteria: (i) a general adult (≥18 years) population, either sex, asymptomatic (*population*); (ii) any study using ML techniques to derive predictive models for future cancer risk using clinical and/or demographic and/or basic laboratory data (*concept*); and (iii) original research articles conducted in all settings in any region of the world (*context*).

**Results:** The search returned 627 unique articles, of which 580 articles were excluded because they did not meet the inclusion criteria, were duplicates, or were related to benign neoplasm. Full-text reviews were conducted for 47 articles and a final set of 10 articles were included in this scoping review. These 10 very heterogeneous studies used ML to predict future cancer risk in asymptomatic individuals. Nine out of 10 ML models reported either excellent or good performance.

**Conclusions:** Research gaps that must be addressed in order to deliver validated ML-based models to assist clinical decision-making include: (i) establishing model generalisability through validation in independent cohorts, including those from low- and middle-income countries; (ii) establishing models for all cancer types; (iii) thorough comparisons of ML models with best available clinical tools to ensure transparency of their potential clinical utility; and (iv) comparisons of different methods on the same cohort to reveal important information about model generalisability and performance.


## ARTICLE SUMMARY

### Strengths and limitations of this study

- This study used the population, concept, and context scoping review approach to explore the machine learning techniques used to derive predictive models for future cancer risk using basic clinical and/or demographic and/or laboratory data (*concept*) in asymptomatic adults ≥18 years (*population*) in all settings in any region of the world (*context*).

For peer review only

- Although the ML methodologies were heterogeneous, the standard use of the area under the receiver operating characteristics curve (AUC) metric to evaluate model performance allowed comparisons of different ML techniques with each other.
- This scoping review is limited to papers published in English between 2011and 2020.

## INTRODUCTION

Cancer remains a leading cause of morbidity and mortality, with an estimated 1.8 million new cases and 0.6 million deaths in the US in 2019 and approximately 367,000 new cases and 165,000 cancer deaths in the UK each year between 2015 and 2017.[1 2] Annual death rates only modestly decreased (1.4% and 1.8% in women and men, respectively) between 2012 and 2016, despite significant research.[1] Cancer cases also continue to increase, not least due to increased life expectancy, which increases the risk of developing cancer.[3]

Early cancer diagnosis is associated with significantly higher survival rate and lower mortality and associated costs. Early-stage cancers require less complex treatment regimens and reduced hospital utilization, resulting in reduced healthcare costs, whereas late-stage cancers require complex multimodal management, several rounds of extremely expensive drugs over significant periods of time, and the treatment of recurrences, equating to a staggering economic burden. Therefore, the importance of early diagnosis cannot be overestimated.[4-6]

Survival rates significantly improve if cancer is diagnosed at stage I or II compared with later stages (stage III and IV),[7 8] as once the cancer has metastasised, it becomes difficult to treat with radiotherapy or surgery, leading to treatment failure and death. For example, five-year survival rates for women diagnosed with localised breast or ovarian cancer are 99% and 92% compared to 27% and 29% for metastatic disease, respectively.[1] A report by Cancer Research UK indicated that, in the UK, the ten-year survival proportions of patients with eight cancers (combined) were around 80% for stage I and stage II detection (breast, bladder, ovarian, colorectal, uterine, testicular, and cervical cancer and malignant melanoma) but only 26% for cancers detected at later stages, notably lung cancer (stage III and IV).[9]


For peer review only - http://bmjopen.bmj.com/site/about/guidelines.xhtml

Globally, treatment for early-stage cancer confer significant cost-saving benefits. In the US, during the first 24 months after diagnosis, there is an increase in cancer treatment costs with stage: US$72,000 for stage 0, US$97,000 for stage I/II, US$159,000 for stage III, and $182,000 for stage IV.[10] An estimate of the cost savings from early cancer diagnosis is 26 billion US dollars per annum in the US alone.[11] Similarly, in the UK, early diagnosis of colorectal, ovarian, and lung cancer in England alone could provide savings of over £44 million and benefit nearly 11,000 patients.[12]

**Current approaches to diagnose incident cancer**

One approach to the early detection of cancer is population-wide screening, which aims to find asymptomatic individuals so that they can be promptly referred for treatment. Examples include mammography for breast cancer, cervical screening for cervical cancer, and faecal occult blood testing or sigmoidoscopy for colorectal cancer.[13] There are three examples of national screening programs in UK (bowel, breast, and cervical cancer screening programs[14]) and two in the US: the Colorectal Cancer Control Program (CRCCP) and the National Breast and Cervical Cancer Early Detection Program (NBCCEDP).[15] However, significant proportions of individuals eligible for these programs do not participate (for example through fear or not prioritizing time to attend for screening),[16] and comprehensive screening programs are costly to implement, especially in resource-poor settings or low- and middle-income countries. Other approaches include public health campaigns to encourage individuals experiencing particular symptoms such as weight loss, anorexia, and fatigue to visit their family doctors.[17] However, patient help-seeking around cancer is complex, multi-staged, and often leads to long delays of weeks or even months.[18] Patients find it hard to interpret and recognise symptoms, with fears of embarrassment and having a potentially fatal or painful condition contributing to long and avoidable delays in help-seeking from health professionals.[18 19] Patients often do not seek help from health professionals for early cancer symptoms, notably from general family physicians, for many reasons including a complex mix of fear, worry, and of 'wasting' health professionals' time[19] or due to the high costs of medical care, a lack of health insurance, or time constraints.[20]

**Detecting future risk of cancer by modelling data**

Screening approaches represent a patient identification (or "phenotyping" problem) that aims to detect whether the individual has cancer at a particular point in time. However, the ultimate goal of cancer prediction is to determine whether an individual will develop cancer at some point in the future. A

For peer review only

simple approach is to stratify populations according to the presence and absence of risk factors, which have been extensively characterised for most cancer types through epidemiological studies over many decades. For example, age, gender, ethnicity, family history, and lifestyle factors are well-established risk factors for many types of cancer.[21] The cancer prediction problem can either be regarded as a supervised learning problem where the input variables are clinical-demographic variables and the output variable is the probability of developing cancer at some point in the future or as a binary classification problem to determine whether or not a patient will develop cancer at a specific point in time.

**Big data and machine learning for medical prediction models**

Advances in digital medicine and computational science have altered the landscape of data available for cancer risk prediction models. For example, in the data-driven healthcare era, there is an increasing amount of “big” medical data, as most individuals have had interactions with the healthcare system where data is collected in the form of electronic health records (EHRs), which are systematic collections of longitudinal patient health data collected in real time.[22 23] Such large datasets provide powerful new opportunities to develop and refine predictive models and to explore potentially unknown predictor variables.[22] Leveraging often massive amounts of data generated from large populations, much of which may be unstructured, and building optimal models requires the exploitation of advanced computational tools and supporting infrastructure. Machine learning (ML) is a branch of artificial intelligence (AI) and an extension of traditional statistical techniques that uses computational resources to detect underlying patterns in high-dimensional data, and it is increasingly being used in different areas of medicine requiring predictions.[24] For example, ML has successfully been used with EHR data to predict incident hypertension[25] and incident chronic kidney disease,[26] and wider popular uses of ML in medicine include the automatic interpretation of medical images such as in radiology[27] and histopathology[28] images.

**A brief description of machine learning**

A comprehensive description of ML models is beyond the scope of this scoping review. However, relevant ML techniques relate to the problem of *learning* from data samples (e.g., EHR data) rather than being pre-programmed with existing knowledge or rules. ML models can either be supervised (i.e., where the data are labelled and the algorithm uses these data to learn to predict the output) or



For peer review only - http://bmjopen.bmj.com/site/about/guidelines.xhtml

unsupervised (i.e., where the data are unlabelled and the algorithm learns a structure inherent in the data).[29] The cancer prediction problem is therefore a supervised problem; examples are provided as inputs (or features) such as cancer risk factors like age, history, ethnicity, or blood count parameters and outputs (or labels) such as whether or not the individual subsequently develops cancer. A variety of available algorithms learn the best way to map the features to the labels by learning from the observations.[30 31] The resulting model, ideally, will then be able to generalise the information so that it can be applied with high precision to new and unseen data.[30 31]

Some of the main supervised ML models used in medical applications include decision trees (DTs; and their adaptation, random forests (RFs)), support vector machines (SVMs), and artificial neural networks (ANNs).[30 31] DTs produce an output similar to a flow chart formed from feature nodes (risk variables) that best discriminate between different labels (future cancer occurrence) to split the tree.[30 31] In this way, new cases can be assessed by traversing the tree based on the feature values to determine the output for that example.[30 31] Decision trees are easy to interpret, since users are usually able to visualise the steps leading to a particular classification, which may be useful in a clinical setting where experts might wish to see how a particular decision was made.[30 31] In RFs, several trees are built using subsets of data and features, with predictions decided based on majority voting after the example is assessed with respect to all the constructed trees.[30 31]

In SVMs, each feature (risk factor) is mapped into a higher-dimensional space and the hyperplane that optimally separates the output (future cancer occurrence) modelled.[30 31] SVMs tend to generalise well to unseen data and work well with complex (multidimensional) data but can be hard to interpret.[30 31]

ANNs are inspired by the neural connections in the human brain and are developed by creating nodes (neurons) that weight certain features and produce an output value.[30 31] By layering nodes in between the input layer (features; cancer risk factors) and output layer (label; future cancer occurrence) and modifying the weights during learning through a process called back-propagation, the resulting model forms a prediction for unseen data when one of the nodes in the output layer is positive.[30 31] The terms “deep neural network” and “deep learning” are applied to ANNs with large numbers of layers.[30 31] While proving extremely powerful across a range of applications, ANNs can be computationally very expensive and the way in which they classify (i.e., the intermediate “hidden”


For peer review only - http://bmjopen.bmj.com/site/about/guidelines.xhtml

layers) is opaque, making it difficult to determine exactly how they performed the classification problem.[30 31]

**Rationale for performing a scoping review**

This was a scoping review of studies using supervised ML techniques to predict the future risk of developing cancer or specific cancers within a general asymptomatic adult (≥18 years) population using clinical and/or demographic and/or basic laboratory data (e.g., complete blood counts) that are likely to be readily available within the primary care setting. This approach therefore allowed to: (i) identify the types of evidence available; (ii) clarify key concepts and definitions; (iii) examine how research is currently being conducted; and (iv) to identify knowledge gaps.[32]

**OBJECTIVES**

The objective of this study was to perform a scoping review and to synthesize knowledge of the nature and effects of current ML techniques for early cancer detection in asymptomatic adults. The scoping review was guided by the following research questions:

(i) Which, if any, ML methods are being developed for cancer risk prediction in asymptomatic individuals in the community?

(ii) How do these models perform compare to each other?

(iii) Which research or knowledge gaps need to be addressed in order to advance the field?


For peer review only - http://bmjopen.bmj.com/site/about/guidelines.xhtml

**METHODS**

**Inclusion and exclusion criteria**

Therefore, using the population, concept, and context approach,[33] the inclusion criteria were: (i) general adult (≥18 years) population, either sex, asymptomatic (*population*); (ii) any study using ML techniques to derive predictive models for future cancer risk using clinical and/or demographic and/or basic laboratory data carried out prior to August 7, 2020 (*concept*); and (iii) original research articles conducted in all settings in any region of the world (*context*).

For the purposes of this study, and recognizing that 'machine learning' algorithms fall along a continuum with statistical techniques,[34] all modelling approaches were included were defined as machine learning in the respective papers (such as logistic regression).

Exclusion criteria were any ML model used to predict future events in patients with pre-existing or symptoms of cancer; ML models developed using specialised tests such as genetic profiling or imaging tests not generally available in the community; unsupervised ML models; and studies not written in English.

**Literature search**

To identify relevant studies, PubMed was searched using the search string:

("Cancer" Or "Cancers" OR "Oncology") AND ("Machine Learning" OR "ML" OR "Data Mining" OR "Decision Support System" OR "Clinical Support System" OR "Classification" OR "Regression" OR "Support vector machines" OR "Gaussian process" OR "Neural networks" OR "Logical learning" OR "Bayesian network" OR "linear model") AND ("prognosis" OR "prognostic estimate" OR "predictor" OR "prediction" OR "model" OR "diagnosis" OR "diagnostic"). This search was supplemented with manual searching of the references and citations of previously published studies. All abstracts identified by the initial search were screened for inclusion and checked for accuracy. For the included studies, data were extracted from full papers. In instances where more information was required to determine inclusion, the full text of the article was retrieved and assessed against the eligibility criteria.

**Assessment metric**

The strength of the predictive ability of the included models was assessed using AUC (area under the receiver operating characteristics curve) data, a valid measure for evaluating classification



For peer review only - http://bmjopen.bmj.com/site/about/guidelines.xhtml

199 algorithms,[35] where an AUC of 0.90-1 = excellent, 0.80-0.89 = good, 0.70-0.79 = fair, 0.60-0.69 = poor, and 0.50-0.59 = fail to describe model performance.[36]

For peer review only


For peer review only - http://bmjopen.bmj.com/site/about/guidelines.xhtml

## RESULTS

### Main Findings

*Identified risk models*

Using the search strategy, 627 initial studies were identified where 10 studies met the inclusion criteria (Table 1; Figure 1).[31 37-46] The most common reasons for exclusion of studies were: (i) models were derived to predict prognosis or responses to therapy in patients with pre-existing cancer; and/or (ii) the studies used features other than clinical and/or demographic and/or basic laboratory data, such as genetic biomarkers. All studies were retrospective cohort or case-control studies conducted between 2011 and 2020, with 8 out of 10 studies completed in the last two years. Eight studies were conducted in the USA and two in Taiwan. One model was built for breast cancer, three for colorectal cancer, one for lung cancer, one for melanoma, two for non-melanoma skin cancer, one for pancreatic cancer, and one a general cancer prediction model. Two studies performed external validations of a previously developed colorectal cancer prediction model (Table 1).[38 42]

*Development of the risk models*

The models developed in the studies employed a wide range of ML techniques. Two studies compared different modelling approaches on the same dataset,[40 43] while the other eight developed a model using a single approach. The following ML approaches were used: ANNs (8 out of 10 studies), logistic regression (2/10 studies), Gaussian naïve Bayes (1 out of 10 studies), Bayesian network inference (1/10 studies), DTs (1/10 studies) and RFs (2/10 studies), linear discriminant analysis (1/10 studies), and SVMs (1/10 studies) (Table 1). Data for training and testing were from medical insurance databases (4/10 studies), EHR data repositories (3/10 studies), surveys (2/10 studies), or represented a retrospective analysis of prospectively collected data from a clinical trial (1/10 studies).

As a result of the diverse cancer types being modelled, study aims, and the available data, a range of different predictors, features, and/or risk factors were included the developed predictive models, which can be grouped into the following categories: (1) patient demographic data: e.g., age, gender, ethnicity, family history; (2) social and lifestyle data: e.g., cigarette smoking and intensity of exercise; (3) comorbidities: e.g., diabetes mellitus, hypertension, congestive heart failure, and chronic obstructive pulmonary disease; (4) clinical and practice data: e.g., Anatomical Therapeutic Chemical (WHO-ATC) prescription codes and clinical encounters; and (5) laboratory tests: e.g., complete blood


For peer review only - http://bmjopen.bmj.com/site/about/guidelines.xhtml
For peer review only

231 count (Table 1). The models that automatically extracted features from EHR records used features
232 that were not always explicitly defined in the respective articles.

For peer review only


For peer review only - http://bmjopen.bmj.com/site/about/guidelines.xhtml

**Table 1.** Summary of studies investigating ML approaches for early cancer detection.

| Type of cancer | Reference | Year | Country | Method | Sample | Input | Validation | Performance | Model performance | Notes |
|---|---|---|---|---|---|---|---|---|---|---|
| Breast | Stark [43] | 2019 | USA | Logistic regression, Gaussian naive Bayes, decision tree, linear discriminant analysis, support vector machine, and feed-forward artificial neural network | 1343 breast cancer and 63,396 non-breast cancer cases (PLCO dataset) | Age, age at menarche, age at first live birth, number of first-degree relatives who have had breast cancer, ethnicity, age at menopause, an indicator of current hormone usage, number of years of hormone usage, BMI, pack years of cigarettes smoked, years of birth control usage, number of live births, and an indicator of personal prior history of cancer | 20% testing data (269 breast cancer and 12,679 non-breast cancer cases) | LR 0.61 (0.58-0.65); NB 0.59 (0.56-0.62); DT 0.51 (0.50-0.52); LDA 0.61 (0.58-0.65); SVM 0.52 (0.48-0.55); NN 0.61 (0.57-0.64) | Fail - poor | At an 0.05 level, the logistic regression, linear discriminant analysis, and neural network with the broader set of inputs were all significantly stronger than the BCRAT |
| Colorectal cancer | Hornbrook [38] | 2017 | USA | ColonFlag ML model | 17,095 US community-based insured adults (16,195 controls, 900 cases) (insurance data) | Age, gender, and blood count panel parameters | Study was a validation of a previously derived model [47] | AUC 0.80 (0.79-0.82) | Good | |
| Colorectal | Wang [45] | 2019 | Taiwan | Convolutional neural network | 10,185 with CRC, 47,967 controls | ICD-9-CM diagnostic codes, World Health Organization- | 5-fold cross-validation | AUC 0.92 | Excellent | |

For peer review only


For peer review only - http://bmjopen.bmj.com/site/about/guidelines.xhtml

| | | | | | | | | | | |
|---|---|---|---|---|---|---|---|---|---|---|
| | | | | | (insurance data) | Anatomical Therapeutic Chemical (WHO-ATC) prescription codes | | | | |
| Colorectal cancer | Schneider [42] | 2020 | USA | ColonFlag ML model | 308,721 insurance health plan members (insurance data) | Age, gender, and blood count panel parameters | Study was a validation of a previously derived model [47] | AUC 0.78 (95% CI 0.77-0.78) | Good | The algorithm's accuracy decreased with the time interval between blood test result and CRC diagnosis |
| General | Miotto [39] | 2016 | USA | Deep neural network and random forests | Model training on 704,587, testing on 76,214 (EHR data) | Features extracted from EHR records | Testing on 76,214 | Colorectal cancer AUC 0.89, liver cancer 0.89, prostate cancer 0.86 | Good | Outperformed RawFeat and PCA |
| Lung | Hart [37] | 2018 | USA | Artificial neural network | 1997-2015 National Health Interview Survey adult data; 648 cancer and 488,418 non-cancer cases (survey data) | Gender, age, BMI, diabetes, smoking status, emphysema, asthma, ethnicity, Hispanic ethnicity, hypertension, heart diseases, vigorous exercise habits, and history of stroke | 30% of data; 195 lung cancer cases and 146,524 never cancer cases | AUC 0.86 (training; 95% CI 0.85-0.88) and 0.86 (validation; 95% CI 0.84-0.89) | Good | Random forests and SVM also applied which trained well (RF AUC of 1.00 (95% CI 1.00- 1.00) and SVM AUC of 0.96 (95% CI 0.95-0.97). However, not generalisable: AUC SVM 0.55 (95% CI 0.51-0.58); AUC RF 0.81 (95% CI 0.78-0.84). |


For peer review only - http://bmjopen.bmj.com/site/about/guidelines.xhtml

| | | | | | | | | | | |
|---|---|---|---|---|---|---|---|---|---|---|
| Melanoma | Richter [40] | 2019 | USA | LR, RF, XGBoost | 4,061,172 patients, 10,129 with melanoma (EHR data) | Features extracted from EHR records | 5-fold cross-validation | AUC LR 0.76; AUC RF 0.69; AUC XGBoost 0.80 | Poor - Good | Smaller amounts of data improved the AUCs |
| Non-melanoma skin cancer | Roffman [41] | 2018 | USA | Artificial neural network | 1997–2015 NHIS adult survey data, 2,056 NMSC and 460,574 non-cancer cases (survey data) | Gender, age, BMI, diabetes, smoking status, emphysema, asthma, ethnicity, Hispanic ethnicity, hypertension, heart diseases, vigorous exercise habits, and history of stroke | 30% for validation (752 NMSC cases and 138,172 never cancer cases) | AUC values of 0.81 (training, 95% CI 0.80–0.82) and 0.81 (validation, 95% CI 0.79–0.82) | Good | |
| Non-melanoma skin cancer | Wang [44] | 2019 | Taiwan | Convolutional neural network | 1829 patients with nonmelanoma skin cancer as their first diagnosed cancer and 7665 random controls (insurance data) | Age, sex, ICD-9-CM diagnostic codes, World Health Organization-Anatomical Therapeutic Chemical (WHO-ATC) prescription codes, and the total numbers of clinical encounters | 5-fold cross-validation | AUC 0.89 (0.87-0.91) | Good | |
| Pancreatic | Zhao [44] | 2011 | USA | Bayesian network inference | 98 cases and 14,971 controls (EHR data) | Demographics, lifestyle, symptoms, co-morbidities, and lab test results (20 variables) | Null | 0.91 (0.87-0.95) | Excellent | |



For peer review only - http://bmjopen.bmj.com/site/about/guidelines.xhtml

**Abbreviations:** AUC, area under the curve; BMI, body mass index; LR, logistic regression; NB, Gaussian naive Bayes; DT, decision tree; LDA, linear discriminant analysis; SVM, support vector machine; ANN artificial neural network; RF, random forest; NMSC, non-melanoma skin cancer; ML, machine learning.

For peer review only


For peer review only - http://bmjopen.bmj.com/site/about/guidelines.xhtml

*Discrimination of the risk models*

All studies provided AUC values as an assessment of model performance. The majority of models (7/10)[31 37-42 44] showed “good” performance, two had “excellent” performance,[45 46] and one “failed”.[43] The two models showing excellent performance were the Bayesian network inference model developed by Zhao et al.,[46] which used 20 demographic, lifestyle, symptom, co-morbidity, and lab test results to predict the risk of pancreatic cancer with an AUC of 0.91, and the CRC predictive model developed by Wang et al.,[45] which used a CNN learning on 1929 features (1099 ICD-9 codes and 830 ATC codes). The models that “failed” were the range of models (logistic regression, Gaussian naive Bayes, DT, LDA, SVM, and feed-forward ANN) developed by Stark et al.[43]; however, as discussed below, although these models only had AUCs between 0.51 and 0.61, two of the models compared favourably with the BRCAT clinical risk tool.

*Comparison of the risk models with existing predictive algorithms*

Stark et al.[43] compared their ML models with an existing clinical prediction tool, the Breast Cancer Risk Prediction Tool (BCRAT; https://bcrisktool.cancer.gov/). The BCRAT tools is an implementation of the Gail model,[48] which is a statistical model that estimates five-year breast cancer risk in women without a personal history of breast cancer and without known mutations in high-risk breast cancer genes such as *BRCA1* and *BRCA2*. In the Gail model, patients self-report their current age, age at menarche, age at first live birth, number of first-degree relatives who have had breast cancer, ethnicity, and number of previous breast biopsies, variables which are weighted within the model by logistic regression.[48] In addition, BCRAT uses data on a personal history of atypical hyperplasia, where available. Although the AUC values for the models (logistic regression (LR), naïve Bayes, DTs, linear discriminant analysis (LDA), SVM, and an ANN) tested using a broader set of features than BCRAT were only between 0.51 (DT) and 0.61 (LR, LDA, and ANN), four of the six models (LR, NB, LDA, and ANN) outperformed BCRAT (AUC 0.56). Other metrics were also used to assess model performance (sensitivity, specificity, and precision), which were comparable between the ML algorithms and the BCRAT, and both BCRAT and the ML models had low precision (~2%). Furthermore, when comparing the different ML models, LR and LDA produced higher AUCs than the ANN model, despite the potential for ANNs to better model noisy data and complex non-linear functions.[49] The authors suggested that this might have been due to the limited amount of available training data or the selection of hyperparameters.[43] It was observed that (i) the derived ML models


For peer review only - http://bmjopen.bmj.com/site/about/guidelines.xhtml

using an extended and set of features available in primary care can deliver improvements on current clinical algorithms; (ii) that adding additional features has a greater impact on improving model performance (i.e., higher AUC) rather than simply using more complex models; and (iii) that AUC values must be interpreted in the context of existing methods, such as existing, clinically-used risk prediction models such as the BCRAT or Gail model, rather than in isolation.

In a systematic review of 52 colorectal cancer models predicting future risk of disease in asymptomatic individuals,[50] 37 models reported AUC values, which ranged from 0.65 and 0.75. These included five models that used routine data exclusively and did not include questionnaires or genetic biomarkers. In comparison, the AUC values for ColonFlag,[38 42] an ML model that uses age, gender, and complete blood count (CBC) features to predict the future occurrence of colorectal cancer up to 12 months prior to diagnosis, were 0.78-0.82.

In another systematic review involving 25 risk prediction models for lung cancer that used only epidemiological parameters as input (i.e., no laboratory parameters),[51] AUCs ranged between 0.57 and 0.86, which compares to an AUC of 0.86 (in both training and validation cohorts) for the ANN model developed by Hart et al.[37] In their systematic review of 25 melanoma risk prediction models,[52] Usher-Smith et al. showed in a summary ROC curve that most models had similar discrimination of 0.76, which compares to the highest AUC of 0.80 achieved using XGBoost ML by Richter et al.[40]

## DISCUSSION

### Strengths and limitations of existing ML approaches

The studies reviewed highlight that several different techniques have successfully been used to develop models with generally very good discriminative performance. Other strengths of the studies are the demonstration of how ML can be applied to large-scale insurance and EHR data containing hundreds or thousands of features in order to build predictive models.

However, the above survey also highlights a number of gaps in the application of ML to predicting the risk of future cancer in asymptomatic individuals. These can be divided into those relating to: (i) study populations; (ii) model types and comparisons; and (iii) model validation and comparisons.


For peer review only - http://bmjopen.bmj.com/site/about/guidelines.xhtml

*Study populations*

To date, ML techniques have only been applied to or validated in datasets from developed countries, representing a fraction of the overall global population and their dietary and lifestyle factors. Given that the aetiology of cancer, risk factors, and genetics differ in different populations,[53] models developed in populations in high-income countries may not be generalisable to those from low- and middle-income countries (LMICs). The development and validation of models in LMICs could have two advantages: first, it would determine the generalizability (and therefore utility) of that model in other populations, better serving the needs of individuals in LMICs; second, disparities between models developed in different geographical settings could provide valuable new information about factors contributing to cancer risk. Generalizing risk prediction models is likely to be challenging, since resource-poor countries often do not have the necessary infrastructure nor the epidemiological research capabilities of institutions in high-income countries.

Furthermore, current ML models predict the risk of a limited number of cancer types. Although breast, colorectal, and lung cancer are the three most common cancers and therefore account for a large proportion of overall cancer burden, it is still important to detect all cancers early. This is especially true for those cancers that are usually silent (asymptomatic) for long periods of time, present late with advanced-stage disease, and for which there are currently no screening programs in place, such as ovarian and pancreatic cancer. Predicting future risk of these cancers could allow closer monitoring of at-risk individuals.

*Model types and comparisons*

A wide variety of ML methodologies have been applied and, despite being applied to the same research problem, this scoping review has not identified a single 'best' method. Two issues arose in studies that compared different ML approaches on the same datasets. First, although different models had similar AUCs during training, not all models generalised well to validation datasets; robust model validation is therefore important to ensure model validity (see below). Second, although in general it is assumed that larger amounts of training data improve model performance,[54] Richter et al.[40] found that equivalent or even better model performance was achievable using reduced datasets (hundreds of thousands vs. millions of datapoints). This might be due to high levels of homogeneity in the "no cancer" class, resulting in fewer instances being required to produce a generalisable model, or as a


For peer review only - http://bmjopen.bmj.com/site/about/guidelines.xhtml

result of overfitting. The requirement for less data for the cancer prediction problem could make ML techniques more accessible to researchers without extensive computing infrastructure and allow smaller datasets to be leveraged for model construction.

*Model validation and comparisons*

With the exception of the two studies evaluating a previously defined algorithm for colorectal cancer, no other study used external validation datasets to assess model generalizability, instead opting for either a single holdout validation sample or 5-fold cross-validation. While useful for assessing overfitting,[55] these approaches do not account for population bias in the training dataset nor differences in other target populations. Studies seeking to develop ML models should seek to validate models in independent populations, recognizing that an advantage of an 'ungeneralisable' model might be insights into cancer risk in other populations. Furthermore, since physicians may code diseases in EHRs differently over time (for instance, due to altered management or incentives), even initially generalisable models may need re-validation over time [23 56].

**Implications for clinical practice**

The ML models described in this scoping review generally show very good performance. So, are any of these models ready for clinical use? The ColonFlag model[38 42] is an example has recently been implemented at Barts Health NHS Trust[57] to identify patients at particularly high risk of CRC, particularly as clinicians struggle to prioritise patients in the backlog created by the COVID-19 pandemic. The ColonFlag model is the only model identified in this scoping review that has undergone extensive external validation in independent datasets.

New ML models need to be contextualised with currently available best clinical practice in order to fully evaluate their potential clinical value. Comparing the relatively poor AUC values of the Stark et al.[43] models with BCRAT revealed that they in fact outperformed it in many cases. In their comparison of their ANN with screening methods for lung cancer such as low-dose CT, chest X-ray, and sputum cytology, Hart et al.[37] noted that (according to sensitivity and specificity) it outperformed most of the other available non-invasive methods. Thorough side-by-side comparisons of newly developed models with other prediction tools would be helpful in establishing future clinical utility.


For peer review only - http://bmjopen.bmj.com/site/about/guidelines.xhtml

**Unanswered questions and future research**

Although the few models that are currently available are methodologically diverse, rarely validated in independent datasets to ensure generalisability, and do not cover all cancer types. Even if ML techniques offer only small improvements in cancer detection rates, these improvements are likely to be of high clinical significance given the large size of the global population with or at high risk of cancer and the high mortality and costs associated with late cancer diagnoses.

However, the scoping review identifies a number of research gaps that will need to be addressed in order to deliver validated ML-based models to assist clinical decision-making. Firstly, future studies must take steps to establish model generalisability through validation in independent cohorts, including those from LMICs. Although the latter may be challenging, it could be argued that even negative generalisability studies might provide an opportunity to learn more about cancer risk factors in different populations. Secondly, the scoping review fails to establish which ML approach best suits the cancer prediction problem but does show that, where possible, side-by-side comparisons of different methods can reveal important information about generalisability as well as performance and that these comparisons are desirable whenever possible. Thirdly, many important cancer types, particularly ‘silent killers’ like ovarian cancer, have currently not been the subject of ML modelling approaches; ML could provide an important, low-cost, non-invasive method to identify individuals at high risk of clinically silent cancers that require closer monitoring. Furthermore, it might not necessarily be true that more data equals improved model performance, which might broaden accessibility of model development to a wider range of clinicians and epidemiologists. Finally, ML models need to be compared to best available clinical tools so that their potential clinical utility is transparent.

**CONCLUSIONS**

In conclusion, this scoping review highlights that the application of ML to cancer prediction is a nascent field, with the majority of the few available studies published in the last two years. Nevertheless, most of ML model performance appears to be good which makes them reliable

For peer review only

approach. We hope that the identified research gaps focus future research efforts to deliver validated ML-based models to assist and improve clinical decision-making.

**Acknowledgment**

The authors would like to thank Dr. Alvina Lai, for her feedback in this scoping review.

**Contributorship statement**

Author AA defined the research question of the scoping review, conducted the literature search, and summarised the findings. Author HK provided academic guidance as domain expert in machine learning for healthcare and revised the draft of the manuscript.

**Competing interests**

Non competing interests

**Funding**

No funding received

**Data sharing statement**

Only public published papers were used. No confidential data

For peer review only

**REFERENCES**

1. Siegel RL, Miller KD, Jemal A. Cancer statistics, 2019. *CA Cancer J Clin* 2019;69(1):7-34. doi: 10.3322/caac.21551

2. Cancer Research UK. Cancer Statistics for the UK 2020 [Available from: https://www.cancerresearchuk.org/health-professional/cancer-statistics-for-the-uk.

3. Cancer Research UK. Why are cancer rates increasing? 2014 [Available from: https://scienceblog.cancerresearchuk.org/2015/02/04/why-are-cancer-rates-increasing/.

4. World Health Organization. Early detection of cancer 2016 [Available from: https://www.who.int/cancer/detection/en/.

5. World Health Organization. Early cancer diagnosis saves lives, cuts treatment costs 2017 [Available from: https://www.who.int/news-room/detail/03-02-2017-early-cancer-diagnosis-saves-lives-cuts-treatment-costs.

6. Phallen J, Sausen M, Adleff V, et al. Direct detection of early-stage cancers using circulating tumor DNA. *Sci Transl Med* 2017;9(403) doi: 10.1126/scitranslmed.aan2415

7. Bannister N, Broggio J. Cancer survival by stage at diagnosis for England (experimental statistics): adults diagnosed 2012, 2013 and 2014 and followed up to 2015. *Produced in collaboration with Public Health England* 2016

8. Canary Foundation. Early Detection Facts and Figures Early Detection Works. California2019.

9. Cancer Research UK. Why is early diagnosis important? 2018 [Available from: https://www.cancerresearchuk.org/about-cancer/cancer-symptoms/why-is-early-diagnosis-important.

10. Blumen H, Fitch K, Polkus V. Comparison of Treatment Costs for Breast Cancer, by Tumor Stage and Type of Service. *Am Health Drug Benefits* 2016;9(1):23-32.

11. Kakushadze Z, Raghubanshi R, Yu W. Estimating cost savings from early cancer diagnosis. *Data* 2017;2(3):30.

12. Cancer Research UK. Saving lives, averting costs 2014 [Available from: https://www.cancerresearchuk.org/sites/default/files/saving_lives_averting_costs.pdf.

13. Weller DP, Campbell C. Uptake in cancer screening programmes: a priority in cancer control. *Br J Cancer* 2009;101 Suppl 2:S55-9. doi: 10.1038/sj.bjc.6605391

For peer review only

14. Cancer Research UK. About cancer screeing 2020 [Available from: https://www.cancerresearchuk.org/about-cancer/screening.

15. Smith RA, Andrews KS, Brooks D, et al. Cancer screening in the United States, 2019: A review of current American Cancer Society guidelines and current issues in cancer screening. *CA Cancer J Clin* 2019;69(3):184-210. doi: 10.3322/caac.21557

16. Joseph DA, King JB, Dowling NF, et al. Vital signs: colorectal cancer screening test use—United States, 2018. *Morbidity and Mortality Weekly Report* 2020;69(10):253.

17. Power E, Wardle J. Change in public awareness of symptoms and perceived barriers to seeing a doctor following Be Clear on Cancer campaigns in England. *British Journal of Cancer* 2015;112(1):S22-S26.

18. Smith LK, Pope C, Botha JL. Patients' help-seeking experiences and delay in cancer presentation: a qualitative synthesis. *Lancet* 2005;366(9488):825-31. doi: 10.1016/S0140-6736(05)67030-4

19. Balasooriya-Smeekens C, Walter FM, Scott S. The role of emotions in time to presentation for symptoms suggestive of cancer: a systematic literature review of quantitative studies. *Psychooncology* 2015;24(12):1594-604. doi: 10.1002/pon.3833

20. Taber JM, Leyva B, Persoskie A. Why do people avoid medical care? A qualitative study using national data. *J Gen Intern Med* 2015;30(3):290-7. doi: 10.1007/s11606-014-3089-1

21. American Cancer Society. Lifetime risk of developing or dying from cancer. 2014

22. Goldstein BA, Navar AM, Pencina MJ, et al. Opportunities and challenges in developing risk prediction models with electronic health records data: a systematic review. *J Am Med Inform Assoc* 2017;24(1):198-208. doi: 10.1093/jamia/ocw042

23. Rose S. Machine Learning for Prediction in Electronic Health Data. *JAMA Netw Open* 2018;1(4):e181404. doi: 10.1001/jamanetworkopen.2018.1404

24. Rajkomar A, Dean J, Kohane I. Machine Learning in Medicine. *N Engl J Med* 2019;380(14):1347-58. doi: 10.1056/NEJMra1814259

25. Ye C, Fu T, Hao S, et al. Prediction of Incident Hypertension Within the Next Year: Prospective Study Using Statewide Electronic Health Records and Machine Learning. *J Med Internet Res* 2018;20(1):e22. doi: 10.2196/jmir.9268

For peer review only

26. Hao S, Fu T, Wu Q, et al. Estimating One-Year Risk of Incident Chronic Kidney Disease: Retrospective Development and Validation Study Using Electronic Medical Record Data From the State of Maine. *JMIR Med Inform* 2017;5(3):e21. doi: 10.2196/medinform.7954

27. Martin Noguerol T, Paulano-Godino F, Martin-Valdivia MT, et al. Strengths, Weaknesses, Opportunities, and Threats Analysis of Artificial Intelligence and Machine Learning Applications in Radiology. *J Am Coll Radiol* 2019;16(9 Pt B):1239-47. doi: 10.1016/j.jacr.2019.05.047

28. Bera K, Schalper KA, Rimm DL, et al. Artificial intelligence in digital pathology - new tools for diagnosis and precision oncology. *Nat Rev Clin Oncol* 2019;16(11):703-15. doi: 10.1038/s41571-019-0252-y

29. Hastie T, Tibshirani R, Friedman J. The elements of statistical learning: data mining, inference, and prediction: Springer Science & Business Media 2009.

30. Kourou K, Exarchos TP, Exarchos KP, et al. Machine learning applications in cancer prognosis and prediction. *Comput Struct Biotechnol J* 2015;13:8-17. doi: 10.1016/j.csbj.2014.11.005

31. Richter AN, Khoshgoftaar TM. A review of statistical and machine learning methods for modeling cancer risk using structured clinical data. *Artif Intell Med* 2018;90:1-14. doi: 10.1016/j.artmed.2018.06.002

32. Munn Z, Peters MDJ, Stern C, et al. Systematic review or scoping review? Guidance for authors when choosing between a systematic or scoping review approach. *BMC Med Res Methodol* 2018;18(1):143. doi: 10.1186/s12874-018-0611-x

33. Peters MD. In no uncertain terms: the importance of a defined objective in scoping reviews. *JBI Database System Rev Implement Rep* 2016;14(2):1-4. doi: 10.11124/jbisrir-2016-2838

34. Beam AL, Kohane IS. Big Data and Machine Learning in Health Care. *JAMA* 2018;319(13):1317-18. doi: 10.1001/jama.2017.18391

35. Waegeman W, De Baets B, Boullart L. ROC analysis in ordinal regression learning. *Pattern Recognition Letters* 2008;29(1):1-9.

36. Tape GE. The Area Under an ROC Curve 2019 [Available from: http://gim.unmc.edu/dxtests/roc3.htm.

37. Hart GR, Roffman DA, Decker R, et al. A multi-parameterized artificial neural network for lung cancer risk prediction. *PLoS One* 2018;13(10):e0205264. doi: 10.1371/journal.pone.0205264

For peer review only



For peer review only - http://bmjopen.bmj.com/site/about/guidelines.xhtml

38. Hornbrook MC, Goshen R, Choman E, et al. Early Colorectal Cancer Detected by Machine Learning Model Using Gender, Age, and Complete Blood Count Data. *Dig Dis Sci* 2017;62(10):2719-27. doi: 10.1007/s10620-017-4722-8

39. Miotto R, Li L, Kidd BA, et al. Deep Patient: An Unsupervised Representation to Predict the Future of Patients from the Electronic Health Records. *Sci Rep* 2016;6:26094. doi: 10.1038/srep26094

40. Richter AN, Khoshgoftaar TM. Efficient learning from big data for cancer risk modeling: A case study with melanoma. *Comput Biol Med* 2019;110:29-39. doi: 10.1016/j.compbiomed.2019.04.039

41. Roffman D, Hart G, Girardi M, et al. Predicting non-melanoma skin cancer via a multi-parameterized artificial neural network. *Sci Rep* 2018;8(1):1701. doi: 10.1038/s41598-018-19907-9

42. Schneider J, Layefsky E, Udaltsova N, et al. Validation of an Algorithm to Identify Patients at Risk for Colorectal Cancer Based on Laboratory Test and Demographic Data in Diverse, Community-Based Population. *Clin Gastroenterol Hepatol* 2020 doi: 10.1016/j.cgh.2020.04.054

43. Stark GF, Hart GR, Nartowt BJ, et al. Predicting breast cancer risk using personal health data and machine learning models. *PLoS One* 2019;14(12):e0226765. doi: 10.1371/journal.pone.0226765

44. Wang HH, Wang YH, Liang CW, et al. Assessment of Deep Learning Using Nonimaging Information and Sequential Medical Records to Develop a Prediction Model for Nonmelanoma Skin Cancer. *JAMA Dermatol* 2019 doi: 10.1001/jamadermatol.2019.2335

45. Wang YH, Nguyen PA, Islam MM, et al. Development of Deep Learning Algorithm for Detection of Colorectal Cancer in EHR Data. *Stud Health Technol Inform* 2019;264:438-41. doi: 10.3233/SHTI190259

46. Zhao D, Weng C. Combining PubMed knowledge and EHR data to develop a weighted bayesian network for pancreatic cancer prediction. *J Biomed Inform* 2011;44(5):859-68. doi: 10.1016/j.jbi.2011.05.004

47. Kinar Y, Kalkstein N, Akiva P, et al. Development and validation of a predictive model for detection of colorectal cancer in primary care by analysis of complete blood counts: a

For peer review only


For peer review only - http://bmjopen.bmj.com/site/about/guidelines.xhtml

518 binational retrospective study. *J Am Med Inform Assoc* 2016;23(5):879-90. doi:
519 10.1093/jamia/ocv195
520 48. Gail MH, Brinton LA, Byar DP, et al. Projecting individualized probabilities of developing breast
521 cancer for white females who are being examined annually. *J Natl Cancer Inst*
522 1989;81(24):1879-86. doi: 10.1093/jnci/81.24.1879
523 49. Lorena AC, Jacintho LF, Siqueira MF, et al. Comparing machine learning classifiers in potential
524 distribution modelling. *Expert Systems with Applications* 2011;38(5):5268-75.
525 50. Usher-Smith JA, Walter FM, Emery JD, et al. Risk Prediction Models for Colorectal Cancer: A
526 Systematic Review. *Cancer Prev Res (Phila)* 2016;9(1):13-26. doi: 10.1158/1940-
527 6207.CAPR-15-0274
528 51. Gray EP, Teare MD, Stevens J, et al. Risk Prediction Models for Lung Cancer: A Systematic
529 Review. *Clin Lung Cancer* 2016;17(2):95-106. doi: 10.1016/j.cllc.2015.11.007
530 52. Usher-Smith JA, Emery J, Kassianos AP, et al. Risk prediction models for melanoma: a
531 systematic review. *Cancer Epidemiol Biomarkers Prev* 2014;23(8):1450-63. doi:
532 10.1158/1055-9965.EPI-14-0295
533 53. Rastogi T, Hildesheim A, Sinha R. Opportunities for cancer epidemiology in developing countries.
534 *Nat Rev Cancer* 2004;4(11):909-17. doi: 10.1038/nrc1475
535 54. van der Ploeg T, Austin PC, Steyerberg EW. Modern modelling techniques are data hungry: a
536 simulation study for predicting dichotomous endpoints. *BMC Med Res Methodol* 2014;14:137.
537 doi: 10.1186/1471-2288-14-137
538 55. A study of cross-validation and bootstrap for accuracy estimation and model selection. Ijcai; 1995.
539 Montreal, Canada.
540 56. Bergquist SL, Brooks GA, Keating NL, et al. Classifying Lung Cancer Severity with Ensemble
541 Machine Learning in Health Care Claims Data. *Proc Mach Learn Res* 2017;68:25-38.
542 57. Downing M. Barts Health using AI to prioritise care for colon cancer patients 2020 [Available from:
543 https://www.bartshealth.nhs.uk/news/barts-health-using-ai-to-prioritise-care-for-high-risk-
544 colon-cancer-patients-8867].

545

546 **FIGURE LEGEND**

547 **Figure 1.** PRISMA flowchart depicting the search strategy.

For peer review only

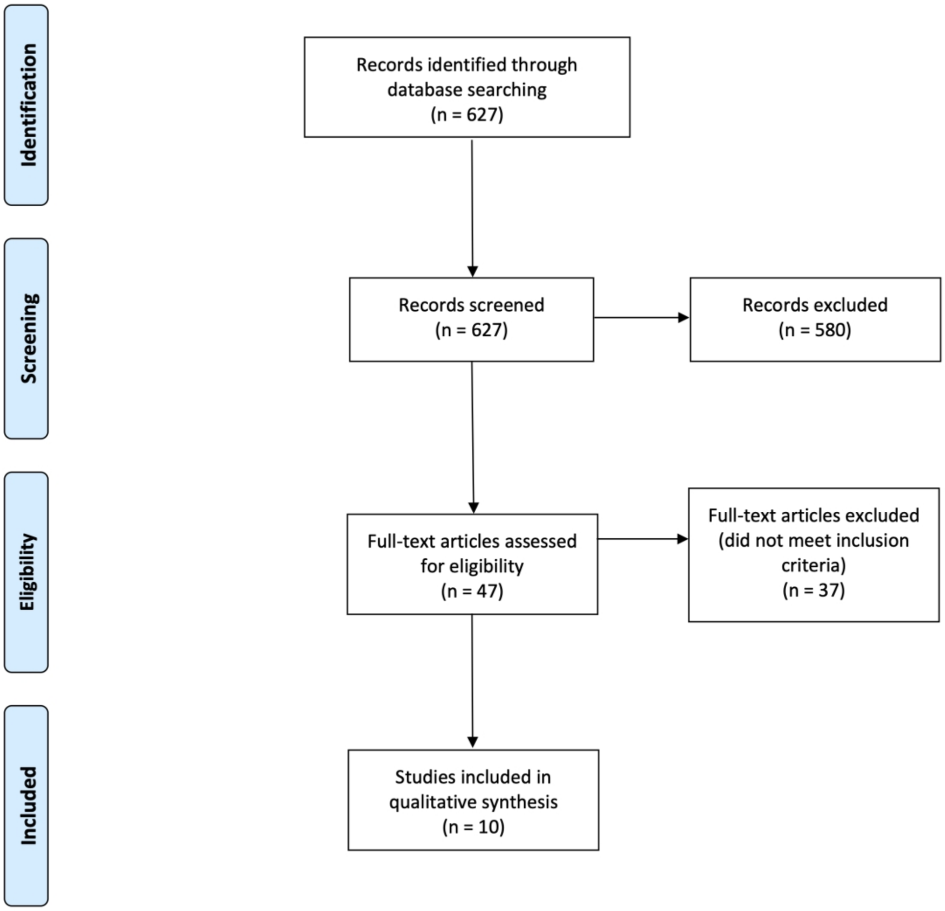


Figure 1. PRISMA flowchart depicting the search strategy.

190x181mm (300 x 300 DPI)

# Preferred Reporting Items for Systematic reviews and Meta-Analyses extension for Scoping Reviews (PRISMA-ScR) Checklist

| SECTION | ITEM | PRISMA-ScR CHECKLIST ITEM | REPORTED ON PAGE # |
|---|---|---|---|
| **TITLE** | | | |
| Title | 1 | Identify the report as a scoping review. | 1 |
| **ABSTRACT** | | | |
| Structured summary | 2 | Provide a structured summary that includes (as applicable): background, objectives, eligibility criteria, sources of evidence, charting methods, results, and conclusions that relate to the review questions and objectives. | 2 |
| **INTRODUCTION** | | | |
| Rationale | 3 | Describe the rationale for the review in the context of what is already known. Explain why the review questions/objectives lend themselves to a scoping review approach. | 7 |
| Objectives | 4 | Provide an explicit statement of the questions and objectives being addressed with reference to their key elements (e.g., population or participants, concepts, and context) or other relevant key elements used to conceptualize the review questions and/or objectives. | 7 |
| **METHODS** | | | |
| Protocol and registration | 5 | Indicate whether a review protocol exists; state if and where it can be accessed (e.g., a Web address); and if available, provide registration information, including the registration number. | NA |
| Eligibility criteria | 6 | Specify characteristics of the sources of evidence used as eligibility criteria (e.g., years considered, language, and publication status), and provide a rationale. | 8 |
| Information sources* | 7 | Describe all information sources in the search (e.g., databases with dates of coverage and contact with authors to identify additional sources), as well as the date the most recent search was executed. | 8 |
| Search | 8 | Present the full electronic search strategy for at least 1 database, including any limits used, such that it could be repeated. | 8 |
| Selection of sources of evidence† | 9 | State the process for selecting sources of evidence (i.e., screening and eligibility) included in the scoping review. | 8 and 10 |
| Data charting process‡ | 10 | Describe the methods of charting data from the included sources of evidence (e.g., calibrated forms or forms that have been tested by the team before their use, and whether data charting was done independently or in duplicate) and any processes for obtaining and confirming data from investigators. | NA |
| Data items | 11 | List and define all variables for which data were sought and any assumptions and simplifications made. | 12, 13, and 14 |
| Critical appraisal of individual sources of evidence§ | 12 | If done, provide a rationale for conducting a critical appraisal of included sources of evidence; describe the methods used and how this information was used in any data synthesis (if appropriate). | NA |

St. Michael's
Inspired Care.
Inspiring Science.

For peer review only - http://bmjopen.bmj.com/site/about/guidelines.xhtml

| SECTION | ITEM | PRISMA-ScR CHECKLIST ITEM | REPORTED ON PAGE # |
|---|---|---|---|
| Synthesis of results | 13 | Describe the methods of handling and summarizing the data that were charted. | 8 and 9 |
| **RESULTS** | | | |
| Selection of sources of evidence | 14 | Give numbers of sources of evidence screened, assessed for eligibility, and included in the review, with reasons for exclusions at each stage, ideally using a flow diagram. | 10 |
| Characteristics of sources of evidence | 15 | For each source of evidence, present characteristics for which data were charted and provide the citations. | 12, 13, and 14 |
| Critical appraisal within sources of evidence | 16 | If done, present data on critical appraisal of included sources of evidence (see item 12). | NA |
| Results of individual sources of evidence | 17 | For each included source of evidence, present the relevant data that were charted that relate to the review questions and objectives. | 12,13 , and 14 |
| Synthesis of results | 18 | Summarize and/or present the charting results as they relate to the review questions and objectives. | 10,11, 16, and 17 |
| **DISCUSSION** | | | |
| Summary of evidence | 19 | Summarize the main results (including an overview of concepts, themes, and types of evidence available), link to the review questions and objectives, and consider the relevance to key groups. | 17-20 |
| Limitations | 20 | Discuss the limitations of the scoping review process. | 17 |
| Conclusions | 21 | Provide a general interpretation of the results with respect to the review questions and objectives, as well as potential implications and/or next steps. | 20-21 |
| **FUNDING** | | | |
| Funding | 22 | Describe sources of funding for the included sources of evidence, as well as sources of funding for the scoping review. Describe the role of the funders of the scoping review. | 21 |

JBI = Joanna Briggs Institute; PRISMA-ScR = Preferred Reporting Items for Systematic reviews and Meta-Analyses extension for Scoping Reviews.

* Where *sources of evidence* (see second footnote) are compiled from, such as bibliographic databases, social media platforms, and Web sites.

† A more inclusive/heterogeneous term used to account for the different types of evidence or data sources (e.g., quantitative and/or qualitative research, expert opinion, and policy documents) that may be eligible in a scoping review as opposed to only studies. This is not to be confused with *information sources* (see first footnote).

‡ The frameworks by Arksey and O'Malley (6) and Levac and colleagues (7) and the JBI guidance (4, 5) refer to the process of data extraction in a scoping review as data charting.

§ The process of systematically examining research evidence to assess its validity, results, and relevance before using it to inform a decision. This term is used for items 12 and 19 instead of "risk of bias" (which is more applicable to systematic reviews of interventions) to include and acknowledge the various sources of evidence that may be used in a scoping review (e.g., quantitative and/or qualitative research, expert opinion, and policy document).

*From:* Tricco AC, Lillie E, Zarin W, O'Brien KK, Colquhoun H, Levac D, et al. PRISMA Extension for Scoping Reviews (PRISMAScR): Checklist and Explanation. Ann Intern Med. 2018;169:467–473. doi: 10.7326/M18-0850.


For peer review only - http://bmjopen.bmj.com/site/about/guidelines.xhtml

BMJ Open

# Predicting the risk of cancer in adults using supervised machine learning: a scoping review

| | |
|---|---|
| Journal: | *BMJ Open* |
| Manuscript ID | bmjopen-2020-047755.R1 |
| Article Type: | Original research |
| Date Submitted by the Author: | 22-Aug-2021 |
| Complete List of Authors: | Alfayez, Asma; University College London; King Abdullah International Medical Research Center<br>Kunz, Holger; University College London<br>Lai, Alvina; University College London |
| <b>Primary Subject Heading</b>: | Health informatics |
| Secondary Subject Heading: | Oncology |
| Keywords: | Information technology < BIOTECHNOLOGY & BIOINFORMATICS, Health informatics < BIOTECHNOLOGY & BIOINFORMATICS, ONCOLOGY |
| | |

SCHOLARONE™
Manuscripts

*I, the Submitting Author has the right to grant and does grant on behalf of all authors of the Work (as defined in the below author licence), an exclusive licence and/or a non-exclusive licence for contributions from authors who are: i) UK Crown employees; ii) where BMJ has agreed a CC-BY licence shall apply, and/or iii) in accordance with the terms applicable for US Federal Government officers or employees acting as part of their official duties; on a worldwide, perpetual, irrevocable, royalty-free basis to BMJ Publishing Group Ltd ("BMJ") its licensees and where the relevant Journal is co-owned by BMJ to the co-owners of the Journal, to publish the Work in this journal and any other BMJ products and to exploit all rights, as set out in our licence.*

*The Submitting Author accepts and understands that any supply made under these terms is made by BMJ to the Submitting Author unless you are acting as an employee on behalf of your employer or a postgraduate student of an affiliated institution which is paying any applicable article publishing charge ("APC") for Open Access articles. Where the Submitting Author wishes to make the Work available on an Open Access basis (and intends to pay the relevant APC), the terms of reuse of such Open Access shall be governed by a Creative Commons licence – details of these licences and which Creative Commons licence will apply to this Work are set out in our licence referred to above.*

*Other than as permitted in any relevant BMJ Author's Self Archiving Policies, I confirm this Work has not been accepted for publication elsewhere, is not being considered for publication elsewhere and does not duplicate material already published. I confirm all authors consent to publication of this Work and authorise the granting of this licence.*

For peer review only

# Predicting the risk of cancer in adults using supervised machine learning: a scoping review

Asma Abdullah Alfayez[1,2,3], Holger Kunz[1], Alvina Grace Lai[1]

[1]Institute of Health Informatics, University College London, London, UK

[2]King Abdullah International Medical Research Center, Riyadh, Saudi Arabia

[3]King Saud bin Abdulaziz University for Health Sciences, Riyadh, Saudi Arabia

***Corresponding author:** Asma Abdullah Alfayez (asma.alfayez.17@ucl.ac.uk); Alvina Grace Lai (alvina.lai@ucl.ac.uk)

**Word count:** 5158

**Keywords:** Machine learning; cancer; prediction; scoping review; artificial intelligence

**Competing interests:** None

For peer review only

## ABSTRACT

**Objectives:** The purpose of this scoping review is to: (i) identify existing supervised machine learning (ML) approaches on the prediction of cancer in asymptomatic adults; (ii) to compare the performance of ML models with each other, and (iii) to identify potential gaps in research.

**Design:** Scoping review using the population, concept, and context approach.

**Search strategy:** PubMed search engine was used from inception through to November 10, 2020 to identify literature meeting following inclusion criteria: (i) a general adult (≥18 years) population, either sex, asymptomatic (*population*); (ii) any study using ML techniques to derive predictive models for future cancer risk using clinical and/or demographic and/or basic laboratory data (*concept*); and (iii) original research articles conducted in all settings in any region of the world (*context*).

**Results:** The search returned 627 unique articles, of which 580 articles were excluded because they did not meet the inclusion criteria, were duplicates, or were related to benign neoplasm. Full-text reviews were conducted for 47 articles and a final set of 10 articles were included in this scoping review. These 10 very heterogeneous studies used ML to predict future cancer risk in asymptomatic individuals. All studies reported area under the receiver operating characteristics curve (AUC) values as metrics of model performance, but no study reported measures of model calibration.

**Conclusions:** Research gaps that must be addressed in order to deliver validated ML-based models to assist clinical decision-making include: (i) establishing model generalisability through validation in independent cohorts, including those from low- and middle-income countries; (ii) establishing models for all cancer types; (iii) thorough comparisons of ML models with best available clinical tools to ensure transparency of their potential clinical utility; (iv) reporting of model calibration performance; and (v) comparisons of different methods on the same cohort to reveal important information about model generalisability and performance.


## ARTICLE SUMMARY

### Strengths and limitations of this study

For peer review only

- This study used a recognised scoping review approach (population, concept, and context) to explore the machine learning techniques used to derive predictive models for future cancer risk.
- Identified studies were not subjected to comprehensive qualitative assessments.
- Only ten studies were identified, making it difficult to draw firm conclusions about their relative performance.
- AUC values alone do not allow for meaningful comparisons of models as they have been trained and evaluated on different datasets under different circumstances and conditions.
- This scoping review is limited to papers published in English until 2020 and only the PubMed search engine was used.

## INTRODUCTION

Cancer remains a leading cause of morbidity and mortality, with an estimated 1.8 million new cases and 0.6 million deaths in the US in 2019 and approximately 367,000 new cases and 165,000 cancer deaths in the UK each year between 2015 and 2017.[1 2] Annual death rates only modestly decreased (1.4% and 1.8% in women and men, respectively) between 2012 and 2016, despite significant research.[1] Cancer cases also continue to increase, not least due to increased life expectancy, which increases the risk of developing cancer.[3]

Early cancer diagnosis is associated with significantly higher survival rate and lower mortality and associated costs. Early-stage cancers require less complex treatment regimens and reduced hospital utilization, resulting in reduced healthcare costs, whereas late-stage cancers require complex multimodal management, several rounds of extremely expensive drugs over significant periods of time, and the treatment of recurrences, equating to a staggering economic burden. Therefore, the importance of early diagnosis cannot be overestimated.[4-6] Treating cancer early has significant cost-saving benefits. In the US, during the first 24 months after diagnosis, there is an increase in cancer treatment costs with stage: US$72,000 for stage 0, US$97,000 for stage I/II, US$159,000 for stage III, and $182,000 for stage IV.[7] An estimate of the cost savings from early cancer diagnosis is 26 billion US dollars per annum in the US alone.[8] Similarly, in the UK, early diagnosis of colorectal, ovarian,


For peer review only - http://bmjopen.bmj.com/site/about/guidelines.xhtml

and lung cancer in England alone could provide savings of over £44 million and benefit nearly 11,000 patients.[9]

Survival rates significantly improve if cancer is diagnosed at stage I or II compared with later stages (stage III and IV),[10 11] as once the cancer has metastasised, it becomes difficult to treat with radiotherapy or surgery, leading to treatment failure and death. For example, five-year survival rates for women diagnosed with localised breast or ovarian cancer are 99% and 92% compared to 27% and 29% for metastatic disease, respectively.[1] A report by Cancer Research UK indicated that, in the UK, the ten-year survival proportions of patients with eight cancers (combined) were around 80% for stage I and stage II detection (breast, bladder, ovarian, colorectal, uterine, testicular, and cervical cancer and malignant melanoma) but only 26% for cancers detected at later stages, notably lung cancer (stage III and IV).[12]

**Current approaches to diagnose incident cancer**

One approach to the early detection of cancer is population-wide screening, which aims to find asymptomatic individuals so that they can be promptly referred for treatment. Examples include mammography for breast cancer, cervical screening for cervical cancer, and faecal occult blood testing or sigmoidoscopy for colorectal cancer.[13] There are three examples of national screening programs in UK (bowel, breast, and cervical cancer screening programs[14]) and two in the US: the Colorectal Cancer Control Program (CRCCP) and the National Breast and Cervical Cancer Early Detection Program (NBCCEDP).[15] However, significant proportions of individuals eligible for these programs do not participate (for example through fear or not prioritizing time to attend for screening),[16] and comprehensive screening programs are costly to implement, especially in resource-poor settings or low- and middle-income countries. Other approaches include public health campaigns to encourage individuals experiencing particular symptoms such as weight loss, anorexia, and fatigue to visit their family doctors.[17] However, patient help-seeking around cancer is complex, multi-staged, and often leads to long delays of weeks or even months.[18] Patients find it hard to interpret and recognise symptoms, with fears of embarrassment and having a potentially fatal or painful condition contributing to long and avoidable delays in help-seeking from health professionals.[18 19] Patients often do not seek help from health professionals for early cancer symptoms, notably from general family physicians, for

For peer review only

many reasons including a complex mix of fear, worry, and of 'wasting' health professionals' time[19] or due to the high costs of medical care, a lack of health insurance, or time constraints.[20]

**Detecting future risk of cancer by modelling data**

Screening approaches represent a patient identification (or "phenotyping" problem) that aims to detect whether the individual has cancer at a particular point in time. However, the ultimate goal of cancer prediction is to determine whether an individual will develop cancer at some point in the future. A simple approach is to stratify populations according to the presence and absence of risk factors, which have been extensively characterised for most cancer types through epidemiological studies over many decades. For example, age, gender, ethnicity, family history, and lifestyle factors are well-established risk factors for many types of cancer.[21] The cancer prediction problem can either be regarded as a regression problem, where the input variables are clinical-demographic variables and the output variable is the probability of developing cancer at some point in the future, or as a binary classification problem to determine whether or not a patient will develop cancer at a specific point in time.

**Big data and machine learning for medical prediction models**

Advances in digital medicine and computational science have altered the landscape of data available for cancer risk prediction models. For example, in the data-driven healthcare era, there is an increasing amount of "big" medical data, as most individuals have had interactions with the healthcare system where data is collected in the form of electronic health records (EHRs), which are systematic collections of longitudinal patient health data collected in real time.[22,23] Such large datasets provide powerful new opportunities to develop and refine predictive models and to explore potentially unknown predictor variables.[22] Leveraging often massive amounts of data generated from large populations, much of which may be unstructured, and building optimal models requires the exploitation of advanced computational tools and supporting infrastructure. Machine learning (ML) is a branch of artificial intelligence (AI) and an extension of traditional statistical techniques that uses computational resources to detect underlying patterns in high-dimensional data, and it is increasingly being used in different areas of medicine requiring predictions.[24] For example, ML has successfully been used with EHR data to predict incident hypertension[25] and incident chronic kidney disease,[26]


For peer review only - http://bmjopen.bmj.com/site/about/guidelines.xhtml

and wider popular uses of ML in medicine include the automatic interpretation of medical images such as in radiology[27] and histopathology[28] images.

**A brief description of machine learning**

A comprehensive description of ML models is beyond the scope of this scoping review. However, relevant ML techniques relate to the problem of *learning* from data samples (e.g., EHR data) rather than being pre-programmed with existing knowledge or rules. ML models can either be supervised (i.e., where the data are labelled and the algorithm uses these data to learn to predict the output) or unsupervised (i.e., where the data are unlabelled and the algorithm learns a structure inherent in the data).[29] The cancer prediction problem is therefore a supervised problem; examples are provided as inputs (or features) such as cancer risk factors like age, history, ethnicity, or blood count parameters and outputs (or labels) such as whether or not the individual subsequently develops cancer. A variety of available algorithms learn the best way to map the features to the labels by learning from the observations.[30,31] The resulting model, ideally, will then be able to generalise the information so that it can be applied with high precision to new and unseen data.[30,31]

Some of the main supervised ML models used in medical applications include decision trees (DTs; and their adaptation, random forests (RFs)), support vector machines (SVMs), and artificial neural networks (ANNs).[30,31] DTs produce an output similar to a flow chart formed from feature nodes (risk variables) that best discriminate between different labels (future cancer occurrence) to split the tree.[30,31] In this way, new cases can be assessed by traversing the tree based on the feature values to determine the output for that example.[30,31] Decision trees are easy to interpret, since users are usually able to visualise the steps leading to a particular classification, which may be useful in a clinical setting where experts might wish to see how a particular decision was made.[30,31] In RFs, several trees are built using subsets of data and features, with predictions decided based on majority voting after the example is assessed with respect to all the constructed trees.[30,31]

In SVMs, each feature (risk factor) is mapped into a higher-dimensional space and the hyperplane that optimally separates the output (future cancer occurrence) modelled.[30,31] SVMs tend to generalise well to unseen data and work well with complex (multidimensional) data but can be hard to interpret.[30,31]

For peer review only



For peer review only - http://bmjopen.bmj.com/site/about/guidelines.xhtml

ANNs are inspired by the neural connections in the human brain and are developed by creating nodes (neurons) that weight certain features and produce an output value.[30 31] By layering nodes in between the input layer (features; cancer risk factors) and output layer (label; future cancer occurrence) and modifying the weights during learning through a process called back-propagation, the resulting model forms a prediction for unseen data when one of the nodes in the output layer is positive.[30 31] The terms "deep neural network" and "deep learning" are applied to ANNs with large numbers of layers.[30 31] While proving extremely powerful across a range of applications, ANNs can be computationally very expensive and the way in which they classify (i.e., the intermediate "hidden" layers) is opaque, making it difficult to determine exactly how they performed the classification problem.[30 31]

**Rationale for performing a scoping review**

Machine learning remains a relatively recent field, so it is unclear exactly to what extent advances have impacted specific healthcare domains. There are currently no extended systematic reviews or scoping reviews on the application of ML to cancer risk prediction in asymptomatic individuals. This prompted us to perform a scoping review of studies using supervised ML techniques to predict the future risk of developing cancer or specific cancers within a general asymptomatic adult (≥18 years) population using clinical and/or demographic and/or basic laboratory data (e.g., complete blood counts) that are likely to be readily available within the primary care setting. This approach therefore allowed to: (i) identify the types of evidence available; (ii) clarify key concepts and definitions; (iii) examine how research is currently being conducted; and (iv) to identify knowledge gaps.[32]

**OBJECTIVES**

The objective of this study was to perform a scoping review and to synthesize knowledge of the nature and effects of current ML techniques for early cancer detection in asymptomatic adults. The scoping review was guided by the following research questions:

(i) Which, if any, ML methods are being developed for cancer risk prediction in asymptomatic individuals in the community?

(ii) How do these models perform compare to each other?

(iii) Which research or knowledge gaps need to be addressed in order to advance the field?


For peer review only - http://bmjopen.bmj.com/site/about/guidelines.xhtml

For peer review only


For peer review only - http://bmjopen.bmj.com/site/about/guidelines.xhtml

## METHODS

### Inclusion and exclusion criteria

We used the population, concept, and context approach[33] with the following inclusion criteria: (i) general adult (≥18 years) population, either sex, asymptomatic (*population*); (ii) any study using ML techniques to derive predictive models for future cancer risk using clinical and/or demographic and/or basic laboratory data carried out prior to August 7, 2020 (*concept*); and (iii) original research articles conducted in all settings in any region of the world (*context*).

For the purposes of this study, and recognizing that 'machine learning' algorithms fall along a continuum with statistical techniques,[34] all modelling approaches were included were defined as machine learning in the respective papers (such as logistic regression).

Exclusion criteria were any ML model used to predict future events in patients with pre-existing or symptoms of cancer; ML models developed using specialised tests such as genetic profiling or imaging tests not generally available in the community; unsupervised ML models; and studies not written in English.

### Literature search

To identify relevant studies, the PubMed database was searched from inception through to November 10, 2020 using the search string: ("Cancer" Or "Cancers" OR "Oncology") AND ("Machine Learning" OR "ML" OR "Data Mining" OR "Decision Support System" OR "Clinical Support System" OR "Classification" OR "Regression" OR "Support vector machines" OR "Gaussian process" OR "Neural networks" OR "Logical learning" OR "Bayesian network" OR "linear model") AND ("prognosis" OR "prognostic estimate" OR "predictor" OR "prediction" OR "model" OR "diagnosis" OR "diagnostic"). This search was supplemented with manual searching of the references and citations of previously published studies. All abstracts identified by the initial search were screened for inclusion and checked for accuracy. For the included studies, data were extracted from full papers. In instances where more information was required to determine inclusion, the full text of the article was retrieved and assessed against the eligibility criteria.

### Study assessment


For peer review only - http://bmjopen.bmj.com/site/about/guidelines.xhtml

The quality of the included studies was assessed using the Newcastle Ottawa Scale (NOS) for observational studies included in the review.[35] The strength of the predictive ability of the included models was assessed using AUC (area under the receiver operating characteristics curve) data, a valid measure for evaluating classification algorithms and one that has been used to compare different algorithms in other meta-analyses.[36 37]

**Patient and public involvement**

This study was not explicitly informed by patient priorities, experiences, and preferences, although the application of predictive models to assess cancer risk would have a direct bearing on identifying those most at risk and implementing investigations in a timely manner. No patients were involved in the design or conduct of the study and since this was a scoping review of the literature, there were no study participants.

For peer review only


For peer review only - http://bmjopen.bmj.com/site/about/guidelines.xhtml

# RESULTS

## Main Findings

*Identified risk models*

Using the search strategy, 627 initial studies were identified where 10 studies met the inclusion criteria (Table 1; Figure 1).[31 38-47] The most common reasons for exclusion of studies were: (i) models were derived to predict outcomes or responses to therapy in patients with pre-existing cancer; and/or (ii) the studies used features other than clinical and/or demographic and/or basic laboratory data, such as genetic biomarkers. All studies were retrospective cohort or case-control studies conducted between 2011 and 2020, with 8 out of 10 studies completed in the last two years. Eight studies were conducted in the USA and two in Taiwan. One model was built for breast cancer, three for colorectal cancer, one for lung cancer, one for melanoma, two for non-melanoma skin cancer, one for pancreatic cancer, and one a general cancer prediction model. Two studies performed external validations of a previously developed colorectal cancer prediction model (Table 1).[39 43] In terms of quality assessment, four studies were graded as "good" quality by the NOS,[39 43 44 46] while six studies were graded as "poor", in all cases due to comparability of cohorts on the basis of the design or analysis adequately controlling for confounders.[31 38 40 42 45 47]

*Development of the risk models*

The models developed in the studies employed a wide range of ML techniques. Two studies compared different modelling approaches on the same dataset,[41 44] while the other eight developed a model using a single approach. The following ML approaches were used: ANNs (8 out of 10 studies), logistic regression (2/10 studies), Gaussian naïve Bayes (1 out of 10 studies), Bayesian network inference (1/10 studies), DTs (1/10 studies) and RFs (2/10 studies), linear discriminant analysis (1/10 studies), and SVMs (1/10 studies) (Table 1). Data for training and testing were from medical insurance databases (4/10 studies), EHR data repositories (3/10 studies), surveys (2/10 studies), or represented a retrospective analysis of prospectively collected data from a clinical trial (1/10 studies).

As a result of the diverse cancer types being modelled, study aims, and the available data, a range of different predictors, features, and/or risk factors were included the developed predictive models, which can be grouped into the following categories: (1) patient demographic data: e.g., age, gender, ethnicity, family history; (2) social and lifestyle data: e.g., cigarette smoking and intensity of exercise;


For peer review only - http://bmjopen.bmj.com/site/about/guidelines.xhtml

(3) comorbidities: e.g., diabetes mellitus, hypertension, congestive heart failure, and chronic obstructive pulmonary disease; (4) clinical and practice data: e.g., Anatomical Therapeutic Chemical (WHO-ATC) prescription codes and clinical encounters; and (5) laboratory tests: e.g., complete blood count (Table 1). The models that automatically extracted features from EHR records used features that were not always explicitly defined in the respective articles.

For peer review only


For peer review only - http://bmjopen.bmj.com/site/about/guidelines.xhtml

**Table 1.** Summary of studies investigating ML approaches for early cancer detection.

| Type of cancer | Reference | Year | Country | Method | Sample | Input | Validation | Performance | NOS | Notes |
|---|---|---|---|---|---|---|---|---|---|---|
| Breast | Stark [44] | 2019 | USA | Logistic regression, Gaussian naive Bayes, decision tree, linear discriminant analysis, support vector machine, and feed-forward artificial neural network | 1343 breast cancer and 63,396 non-breast cancer cases (PLCO dataset) | Age, age at menarche, age at first live birth, number of first-degree relatives who have had breast cancer, ethnicity, age at menopause, an indicator of current hormone usage, number of years of hormone usage, BMI, pack years of cigarettes smoked, years of birth control usage, number of live births, and an indicator of personal prior history of cancer | 20% testing data (269 breast cancer and 12,679 non-breast cancer cases) | LR 0.61 (0.58-0.65); NB 0.59 (0.56-0.62); DT 0.51 (0.50-0.52); LDA 0.61 (0.58-0.65); SVM 0.52 (0.48-0.55); NN 0.61 (0.57-0.64) | 9 (Good) | At an 0.05 level, the logistic regression, linear discriminant analysis, and neural network with the broader set of inputs were all significantly stronger than the BCRAT |
| Colorectal cancer | Hornbrook [39] | 2017 | USA | ColonFlag ML model | 17,095 US community-based insured adults (16,195 controls, 900 cases) (insurance data) | Age, gender, and blood count panel parameters | Study was a validation of a previously derived model [48] | AUC 0.80 (0.79-0.82) | 7 (Good) | |
| Colorectal | Wang [46] | 2019 | Taiwan | Convolutional neural network | 10,185 with CRC, 47,967 controls | ICD-9-CM diagnostic codes, World Health Organization- | 5-fold cross-validation | AUC 0.92 | 7 (Good) | |



For peer review only - http://bmjopen.bmj.com/site/about/guidelines.xhtml

| | | | | | | | | | | |
|---|---|---|---|---|---|---|---|---|---|---|
| | | | | | (insurance data) | Anatomical Therapeutic Chemical (WHO-ATC) prescription codes | | | | |
| Colorectal cancer | Schneider [43] | 2020 | USA | ColonFlag ML model | 308,721 insurance health plan members (insurance data) | Age, gender, and blood count panel parameters | Study was a validation of a previously derived model [48] | AUC 0.78 (95% CI 0.77-0.78) | 8 (Good) | The algorithm's accuracy decreased with the time interval between blood test result and CRC diagnosis |
| General | Miotto [40] | 2016 | USA | Deep neural network and random forests | Model training on 704,587, testing on 76,214 (EHR data) | Features extracted from EHR records | Testing on 76,214 | Colorectal cancer AUC 0.89, liver cancer 0.89, prostate cancer 0.86 | 6 (Poor) | Outperformed RawFeat and PCA |
| Lung | Hart [38] | 2018 | USA | Artificial neural network | 1997-2015 National Health Interview Survey adult data; 648 cancer and 488,418 non-cancer cases (survey data) | Gender, age, BMI, diabetes, smoking status, emphysema, asthma, ethnicity, Hispanic ethnicity, hypertension, heart diseases, vigorous exercise habits, and history of stroke | 30% of data; 195 lung cancer cases and 146,524 never cancer cases | AUC 0.86 (training; 95% CI 0.85-0.88) and 0.86 (validation; 95% CI 0.84-0.89) | 6 (Poor) | Random forests and SVM also applied which trained well (RF AUC of 1.00 (95% CI 1.00- 1.00) and SVM AUC of 0.96 (95% CI 0.95-0.97). However, not generalisable: AUC SVM 0.55 (95% CI 0.51-0.58); AUC RF 0.81 (95% CI 0.78-0.84). |


For peer review only - http://bmjopen.bmj.com/site/about/guidelines.xhtml

| | | | | | | | | | | |
|---|---|---|---|---|---|---|---|---|---|---|
| Melanoma | Richter [41] | 2019 | USA | LR, RF, XGBoost | 4,061,172 patients, 10,129 with melanoma (EHR data) | Features extracted from EHR records | 5-fold cross-validation | AUC LR 0.76; AUC RF 0.69; AUC XGBoost 0.80 | 7 (Poor) | Smaller amounts of data improved the AUCs |
| Non-melanoma skin cancer | Roffman [42] | 2018 | USA | Artificial neural network | 1997–2015 NHIS adult survey data, 2,056 NMSC and 460,574 non-cancer cases (survey data) | Gender, age, BMI, diabetes, smoking status, emphysema, asthma, ethnicity, Hispanic ethnicity, hypertension, heart diseases, vigorous exercise habits, and history of stroke | 30% for validation (752 NMSC cases and 138,172 never cancer cases) | AUC values of 0.81 (training, 95% CI 0.80–0.82) and 0.81 (validation, 95% CI 0.79–0.82) | 6 (Poor) | |
| Non-melanoma skin cancer | Wang [45] | 2019 | Taiwan | Convolutional neural network | 1829 patients with nonmelanoma skin cancer as their first diagnosed cancer and 7665 random controls (insurance data) | Age, sex, ICD-9-CM diagnostic codes, World Health Organization-Anatomical Therapeutic Chemical (WHO-ATC) prescription codes, and the total numbers of clinical encounters | 5-fold cross-validation | AUC 0.89 (0.87-0.91) | 6 (Poor) | |
| Pancreatic | Zhao [47] | 2011 | USA | Bayesian network inference | 98 cases and 14,971 controls (EHR data) | Demographics, lifestyle, symptoms, co-morbidities, and lab test results (20 variables) | Null | 0.91 (0.87-0.95) | 4 (Poor) | |

For peer review only

**Abbreviations:** ANN artificial neural network; AUC, area under the curve; BMI, body mass index; LR, logistic regression; NB, Gaussian naive Bayes; DT, decision tree; LDA, linear discriminant analysis; ML, machine learning; NMSC, non-melanoma skin cancer; NOS, Newcastle Ottawa Scale; RF, random forest; SVM, support vector machine.

For peer review only

*Discrimination and calibration of the risk models*

All studies provided AUC values as an assessment of model performance. Calibration (i.e., whether the risk estimates were accurate), was not assessed in any study. Two models with particularly high AUC values were the Bayesian network inference model developed by Zhao et al.,[47] which used 20 demographic, lifestyle, symptom, co-morbidity, and lab test results to predict the risk of pancreatic cancer with an AUC of 0.91, and the CRC predictive model developed by Wang et al.,[46] which used a CNN learning on 1929 features (1099 ICD-9 codes and 830 ATC codes). Models with particularly low AUC values were the range of models (logistic regression, Gaussian naive Bayes, DT, LDA, SVM, and feed-forward ANN) developed by Stark et al.[44]; however, as discussed below, although these models only had AUCs between 0.51 and 0.61, two of the models compared favourably with the BRCAT clinical risk tool.

*Comparison of the risk models with existing predictive algorithms*

Hundreds of risk prediction models have been published in the literature for every cancer type, and some of these are already used in clinical practice. It is therefore important to understand whether the performance of the newer ML-based cancer risk models is comparable to that of existing predictive algorithms. We therefore specifically examined whether the studies compared their ML algorithms with existing algorithms or, if not, how model performance as described by AUCs compared with other published data, despite the limitations of this approach (see below).

Stark et al.[44] compared their ML models with an existing clinical prediction tool, the Breast Cancer Risk Prediction Tool (BCRAT; https://bcrisktool.cancer.gov/). The BCRAT tools is an implementation of the Gail model,[49] which is a statistical model that estimates five-year breast cancer risk in women without a personal history of breast cancer and without known mutations in high-risk breast cancer genes such as *BRCA1* and *BRCA2*. In the Gail model, patients self-report their current age, age at menarche, age at first live birth, number of first-degree relatives who have had breast cancer, ethnicity, and number of previous breast biopsies, variables which are weighted within the model by logistic regression.[49] In addition, BCRAT uses data on a personal history of atypical hyperplasia, where available. Although the AUC values for the models (logistic regression (LR), naïve Bayes, DTs, linear discriminant analysis (LDA), SVM, and an ANN) tested using a broader set of features than BCRAT were only between 0.51 (DT) and 0.61 (LR, LDA, and ANN), four of the six models (LR, NB, LDA, and ANN) outperformed BCRAT (AUC 0.56). Other metrics were also used to assess model



For peer review only - http://bmjopen.bmj.com/site/about/guidelines.xhtml

performance (sensitivity, specificity, and precision), which were comparable between the ML algorithms and the BCRAT, and both BCRAT and the ML models had low precision (~2%). Furthermore, when comparing the different ML models, LR and LDA produced higher AUCs than the ANN model, despite the potential for ANNs to better model noisy data and complex non-linear functions.[50] The authors suggested that this might have been due to the limited amount of available training data or the selection of hyperparameters.[44] It was observed that (i) the derived ML models using an extended and set of features available in primary care can deliver improvements on current clinical algorithms; (ii) that adding additional features has a greater impact on improving model performance (i.e., higher AUC) rather than simply using more complex models; and (iii) that AUC values must be interpreted in the context of existing methods, such as existing, clinically-used risk prediction models such as the BCRAT or Gail model, rather than in isolation.

In a systematic review of 52 colorectal cancer models predicting future risk of disease in asymptomatic individuals,[51] 37 models reported AUC values, which ranged from 0.65 and 0.75. These included five models that used routine data exclusively and did not include questionnaires or genetic biomarkers. In comparison, the AUC values for ColonFlag,[39 43] an ML model that uses age, gender, and complete blood count (CBC) features to predict the future occurrence of colorectal cancer up to 12 months prior to diagnosis, were 0.78-0.82.

In another systematic review involving 25 risk prediction models for lung cancer that used only epidemiological parameters as input (i.e., no laboratory parameters),[52] AUCs ranged between 0.57 and 0.86, which compares to an AUC of 0.86 (in both training and validation cohorts) for the ANN model developed by Hart et al.[38] In their systematic review of 25 melanoma risk prediction models,[53] Usher-Smith et al. showed in a summary ROC curve that most models had similar discrimination of 0.76, which compares to the highest AUC of 0.80 achieved using XGBoost ML by Richter et al.[41]

## DISCUSSION

### Strengths and limitations of existing ML approaches

The reviewed studies reviewed highlight that several different techniques have successfully been used to develop models and that ML can be applied to large-scale insurance and EHR data containing hundreds or thousands of features in order to build predictive models. However, the survey


For peer review only - http://bmjopen.bmj.com/site/about/guidelines.xhtml

306 also highlights a number of gaps in the application of ML to predicting the risk of future cancer in
307 asymptomatic individuals. These can be divided into those relating to: (i) study populations; (ii) model
308 types and comparisons; and (iii) model validation, comparisons, and calibration.

309 *Study populations*

310 To date, ML techniques have only been applied to or validated in datasets from developed countries,
311 representing a fraction of the overall global population and their dietary and lifestyle factors. Given
312 that the aetiology of cancer, risk factors, and genetics differ in different populations,[54] models
313 developed in populations in high-income countries may not be generalisable to those from low- and
314 middle-income countries (LMICs). The development and validation of models in LMICs could have
315 two advantages: first, it would determine the generalizability (and therefore utility) of that model in
316 other populations, better serving the needs of individuals in LMICs; second, disparities between
317 models developed in different geographical settings could provide valuable new information about
318 factors contributing to cancer risk. Generalizing risk prediction models is likely to be challenging, since
319 resource-poor countries often do not have the necessary infrastructure nor the epidemiological
320 research capabilities of institutions in high-income countries.

321 Furthermore, current ML models predict the risk of a limited number of cancer types. Although breast,
322 colorectal, and lung cancer are the three most common cancers and therefore account for a large
323 proportion of overall cancer burden, it is still important to detect all cancers early. This is especially
324 true for those cancers that are usually silent (asymptomatic) for long periods of time, present late with
325 advanced-stage disease, and for which there are currently no screening programs in place, such as
326 ovarian and pancreatic cancer. Predicting future risk of these cancers could allow closer monitoring of
327 at-risk individuals.

328 *Model types and comparisons*

329 A wide variety of ML methodologies have been applied and, despite being applied to the same
330 research problem, this scoping review has not identified a single 'best' method. Two issues arose in
331 studies that compared different ML approaches on the same datasets. First, although different models
332 had similar AUCs during training, not all models generalised well to validation datasets; robust model
333 validation is therefore important to ensure model validity (see below). Second, although in general it is
334 assumed that larger amounts of training data improve model performance,[55] Richter et al.[41] found that



For peer review only - http://bmjopen.bmj.com/site/about/guidelines.xhtml

equivalent or even better model performance was achievable using reduced datasets (hundreds of thousands vs. millions of datapoints). This might be due to high levels of homogeneity in the "no cancer" class, resulting in fewer instances being required to produce a generalisable model, or as a result of overfitting. Although the requirement for less data for the cancer prediction problem could make ML techniques more accessible to researchers without extensive computing infrastructure and allow smaller datasets to be leveraged for model construction, ML requires over ten-times the amount of data per variable for stable discrimination compared with traditional approaches such as logistic regression.[55] Instead of regarding data requirements as "too high" or "too low", it might be better to consider how much data is required for a particular predictive context. Riley et al.[56] recently provided an implementation of how to calculate the sample size required to develop specific clinical prediction models, which will help researchers prospectively plan their *in silico* experiments and avoid using datasets that are too small for the total number of participants or outcome events.

*Model validation, comparisons, and performance evaluation*

With the exception of the two studies evaluating a previously defined algorithm for colorectal cancer, no other study used external validation datasets to assess model generalizability, instead opting for either a single holdout validation sample or 5-fold cross-validation. While useful for assessing overfitting,[57] these approaches do not account for population bias in the training dataset nor differences in other target populations. Studies seeking to develop ML models should seek to validate models in independent populations, recognizing that an advantage of an 'ungeneralisable' model might be insights into cancer risk in other populations. Furthermore, since physicians may code diseases in EHRs differently over time (for instance, due to altered management or incentives), even initially generalisable models may need re-validation over time.[23 58]

Discrimination (i.e., the ability to distinguish a patient with a high(er) risk of developing cancer from one with a low(er) risk of developing cancer) was measured in every study using the AUC, as is common in the field. However, discrimination is not the only metric of model performance.[59] Another important measure of model performance, particularly for the clinical setting, is calibration; that is, establishing that the risk estimates are accurate.[60] In this setting, this means that the model should not unduly over- or underestimate the risk that a patient will develop cancer; to do so would mean that

For peer review only

a patient might be subjected to investigations and the associated worry of their likelihood of developing cancer (overestimated risk), or, conversely, under-investigated and falsely reassured in the case of underestimated risk. Therefore, a highly discriminatory but poorly calibrated model is likely to have poor clinical utility.

None of the studies reviewed here performed calibration analysis, which is not uncommon in this field. Indeed, in their systematic review of 71 studies using ML for clinical prediction for a wide variety of clinical purposes, Christodoulou et al. reported that 79% of studies failed to address the calibration problem.[37] Therefore, caution must be applied when interpreting and comparing the performance of current ML models based on AUC alone, since is an incomplete measure of performance that must be considered together with methodological aspects such overfitting, measurement error, and population heterogeneity that might influence the estimation of predictive performance.[37 60]

**Implications for clinical practice**

The ML models described in this scoping review generally show high AUC values. So, are any of these models ready for clinical use? The ColonFlag model[39 43] is an example has recently been implemented at Barts Health NHS Trust[61] to identify patients at particularly high risk of CRC, particularly as clinicians struggle to prioritise patients in the backlog created by the COVID-19 pandemic. The ColonFlag model is the only model identified in this scoping review that has undergone extensive external validation in independent datasets.

New ML models need to be contextualised with currently available best clinical practice in order to fully evaluate their potential clinical value. Comparing the relatively poor AUC values of the Stark et al.[44] models with BCRAT revealed that they in fact outperformed it in many cases. In their comparison of their ANN with screening methods for lung cancer such as low-dose CT, chest X-ray, and sputum cytology, Hart et al.[38] noted that (according to sensitivity and specificity) it outperformed most of the other available non-invasive methods. Thorough side-by-side comparisons of newly developed models with other prediction tools would be helpful in establishing future clinical utility.

Finally, this scoping review highlights that model performance should not be evaluated solely on the basis of AUC values but also in terms of other importance performance metrics such as calibration,

For peer review only

without which a model might inaccurately assess risk and therefore prompt inappropriate management.

**Unanswered questions and future research**

The few models that are currently available are methodologically diverse, rarely validated in independent datasets to ensure generalisability, and do not cover all cancer types. Even if ML techniques offer only small improvements in cancer detection rates, these improvements are likely to be of high clinical significance given the large size of the global population with or at high risk of cancer and the high mortality and costs associated with late cancer diagnoses.

However, the scoping review identifies a number of research gaps that will need to be addressed in order to deliver validated ML-based models to assist clinical decision-making. Firstly, future studies must take steps to establish model generalisability through validation in independent cohorts, including those from LMICs. Although the latter may be challenging, it could be argued that even negative generalisability studies might provide an opportunity to learn more about cancer risk factors in different populations. Secondly, the scoping review fails to establish which ML approach best suits the cancer prediction problem but does show that, where possible, side-by-side comparisons of different methods can reveal important information about generalisability as well as performance and that these comparisons are desirable whenever possible. Thirdly, many important cancer types, particularly 'silent killers' like ovarian cancer, have currently not been the subject of ML modelling approaches; ML could provide an important, low-cost, non-invasive method to identify individuals at high risk of clinically silent cancers that require closer monitoring. Fourthly, progress has been made in defining approaches to tailor sample sizes to the specific setting of interest to minimise overfitting and targeting precise estimates of key parameters, and these principles must be applied when testing and validating models to ensure robust model performance. Finally, ML models need to be compared to best available clinical tools so that their potential clinical utility is transparent.

**Limitations of this study**

For peer review only


For peer review only - http://bmjopen.bmj.com/site/about/guidelines.xhtml

Our study has a number of limitations. First, despite recognising the need for a scoping review due to the paucity of literature on the topic, we were only able to identify ten papers meeting the inclusion criteria. It is therefore difficult to draw definitive conclusions about the performance of these models. Furthermore, although AUC values provide an indication of how discriminative the models are, they do not allow for meaningful comparisons of models trained and evaluated on different datasets. Six out of ten studies were defined as poor quality due to a lack of controlling for confounders in the study design, which may have introduced significant bias. Finally, we only search the PubMed database and articles published in English, so some papers in other languages or in databases for non-medical disciplines may have been missed.

For peer review only

## CONCLUSIONS

This scoping review highlights that applying ML to cancer prediction is a promising field provided that the identified issues such as generalisability, validation and clinical applicability, model calibration, and dataset selection are addressed in future studies. We hope that the identified research gaps focus future research efforts to deliver validated ML-based models to assist and improve clinical decision-making

### Contributorship statement

AAA defined the research question of the scoping review, conducted the literature search, and summarised the findings. HK and AGL supervised the research. All authors drafted and revised the manuscript.

### Competing interests

No competing interests.

### Funding

448 AGL is supported by funding from the Wellcome Trust (204841/Z/16/Z), National Institute for Health
449 Research (NIHR) University College London Hospitals Biomedical Research Centre
450 (BRC714/HI/RW/101440), NIHR Great Ormond Street Hospital Biomedical Research Centre
451 (19RX02).

452 **Data sharing statement**

453 Only public published papers were used.

454 **Ethics Approval**

455 Not Applicable. This study does not involve human participants nor animal subjects

For peer review only


For peer review only - http://bmjopen.bmj.com/site/about/guidelines.xhtml

## REFERENCES


1. Siegel RL, Miller KD, Jemal A. Cancer statistics, 2019. *CA Cancer J Clin* 2019;69(1):7-34. doi: 10.3322/caac.21551
2. Cancer Research UK. Cancer Statistics for the UK 2020 [Available from: https://www.cancerresearchuk.org/health-professional/cancer-statistics-for-the-uk].
3. Cancer Research UK. Why are cancer rates increasing? 2014 [Available from: https://scienceblog.cancerresearchuk.org/2015/02/04/why-are-cancer-rates-increasing/].
4. World Health Organization. Early detection of cancer 2016 [Available from: https://www.who.int/cancer/detection/en/].
5. World Health Organization. Early cancer diagnosis saves lives, cuts treatment costs 2017 [Available from: https://www.who.int/news-room/detail/03-02-2017-early-cancer-diagnosis-saves-lives-cuts-treatment-costs].
6. Phallen J, Sausen M, Adleff V, et al. Direct detection of early-stage cancers using circulating tumor DNA. *Sci Transl Med* 2017;9(403) doi: 10.1126/scitranslmed.aan2415
7. Blumen H, Fitch K, Polkus V. Comparison of Treatment Costs for Breast Cancer, by Tumor Stage and Type of Service. *Am Health Drug Benefits* 2016;9(1):23-32.
8. Kakushadze Z, Raghubanshi R, Yu W. Estimating cost savings from early cancer diagnosis. *Data* 2017;2(3):30.
9. Cancer Research UK. Saving lives, averting costs 2014 [Available from: https://www.cancerresearchuk.org/sites/default/files/saving_lives_averting_costs.pdf].
10. Bannister N, Broggio J. Cancer survival by stage at diagnosis for England (experimental statistics): adults diagnosed 2012, 2013 and 2014 and followed up to 2015. *Produced in collaboration with Public Health England* 2016
11. Canary Foundation. Early Detection Facts and Figures Early Detection Works. California2019.
12. Cancer Research UK. Why is early diagnosis important? 2018 [Available from: https://www.cancerresearchuk.org/about-cancer/cancer-symptoms/why-is-early-diagnosis-important].
13. Weller DP, Campbell C. Uptake in cancer screening programmes: a priority in cancer control. *Br J Cancer* 2009;101 Suppl 2:S55-9. doi: 10.1038/sj.bjc.6605391




For peer review only - http://bmjopen.bmj.com/site/about/guidelines.xhtml

14. Cancer Research UK. About cancer screeing 2020 [Available from: https://www.cancerresearchuk.org/about-cancer/screening].

15. Smith RA, Andrews KS, Brooks D, et al. Cancer screening in the United States, 2019: A review of current American Cancer Society guidelines and current issues in cancer screening. *CA Cancer J Clin* 2019;69(3):184-210. doi: 10.3322/caac.21557

16. Joseph DA, King JB, Dowling NF, et al. Vital signs: colorectal cancer screening test use—United States, 2018. *Morbidity and Mortality Weekly Report* 2020;69(10):253.

17. Power E, Wardle J. Change in public awareness of symptoms and perceived barriers to seeing a doctor following Be Clear on Cancer campaigns in England. *British Journal of Cancer* 2015;112(1):S22-S26.

18. Smith LK, Pope C, Botha JL. Patients' help-seeking experiences and delay in cancer presentation: a qualitative synthesis. *Lancet* 2005;366(9488):825-31. doi: 10.1016/S0140-6736(05)67030-4

19. Balasooriya-Smeekens C, Walter FM, Scott S. The role of emotions in time to presentation for symptoms suggestive of cancer: a systematic literature review of quantitative studies. *Psychooncology* 2015;24(12):1594-604. doi: 10.1002/pon.3833

20. Taber JM, Leyva B, Persoskie A. Why do people avoid medical care? A qualitative study using national data. *J Gen Intern Med* 2015;30(3):290-7. doi: 10.1007/s11606-014-3089-1

21. American Cancer Society. Lifetime risk of developing or dying from cancer. 2014

22. Goldstein BA, Navar AM, Pencina MJ, et al. Opportunities and challenges in developing risk prediction models with electronic health records data: a systematic review. *J Am Med Inform Assoc* 2017;24(1):198-208. doi: 10.1093/jamia/ocw042

23. Rose S. Machine Learning for Prediction in Electronic Health Data. *JAMA Netw Open* 2018;1(4):e181404. doi: 10.1001/jamanetworkopen.2018.1404

24. Rajkomar A, Dean J, Kohane I. Machine Learning in Medicine. *N Engl J Med* 2019;380(14):1347-58. doi: 10.1056/NEJMra1814259

25. Ye C, Fu T, Hao S, et al. Prediction of Incident Hypertension Within the Next Year: Prospective Study Using Statewide Electronic Health Records and Machine Learning. *J Med Internet Res* 2018;20(1):e22. doi: 10.2196/jmir.9268

For peer review only



For peer review only - http://bmjopen.bmj.com/site/about/guidelines.xhtml

26. Hao S, Fu T, Wu Q, et al. Estimating One-Year Risk of Incident Chronic Kidney Disease: Retrospective Development and Validation Study Using Electronic Medical Record Data From the State of Maine. *JMIR Med Inform* 2017;5(3):e21. doi: 10.2196/medinform.7954

27. Martin Noguerol T, Paulano-Godino F, Martin-Valdivia MT, et al. Strengths, Weaknesses, Opportunities, and Threats Analysis of Artificial Intelligence and Machine Learning Applications in Radiology. *J Am Coll Radiol* 2019;16(9 Pt B):1239-47. doi: 10.1016/j.jacr.2019.05.047

28. Bera K, Schalper KA, Rimm DL, et al. Artificial intelligence in digital pathology - new tools for diagnosis and precision oncology. *Nat Rev Clin Oncol* 2019;16(11):703-15. doi: 10.1038/s41571-019-0252-y

29. Hastie T, Tibshirani R, Friedman J. The elements of statistical learning: data mining, inference, and prediction: Springer Science & Business Media 2009.

30. Kourou K, Exarchos TP, Exarchos KP, et al. Machine learning applications in cancer prognosis and prediction. *Comput Struct Biotechnol J* 2015;13:8-17. doi: 10.1016/j.csbj.2014.11.005

31. Richter AN, Khoshgoftaar TM. A review of statistical and machine learning methods for modeling cancer risk using structured clinical data. *Artif Intell Med* 2018;90:1-14. doi: 10.1016/j.artmed.2018.06.002

32. Munn Z, Peters MDJ, Stern C, et al. Systematic review or scoping review? Guidance for authors when choosing between a systematic or scoping review approach. *BMC Med Res Methodol* 2018;18(1):143. doi: 10.1186/s12874-018-0611-x

33. Peters MD. In no uncertain terms: the importance of a defined objective in scoping reviews. *JBI Database System Rev Implement Rep* 2016;14(2):1-4. doi: 10.11124/jbisrir-2016-2838

34. Beam AL, Kohane IS. Big Data and Machine Learning in Health Care. *JAMA* 2018;319(13):1317-18. doi: 10.1001/jama.2017.18391

35. Stang A. Critical evaluation of the Newcastle-Ottawa scale for the assessment of the quality of nonrandomized studies in meta-analyses. *Eur J Epidemiol* 2010;25(9):603-5. doi: 10.1007/s10654-010-9491-z

36. Waegeman W, De Baets B, Boullart L. ROC analysis in ordinal regression learning. *Pattern Recognition Letters* 2008;29(1):1-9.

For peer review only



For peer review only - http://bmjopen.bmj.com/site/about/guidelines.xhtml

37. Christodoulou E, Ma J, Collins GS, et al. A systematic review shows no performance benefit of machine learning over logistic regression for clinical prediction models. *J Clin Epidemiol* 2019;110:12-22. doi: 10.1016/j.jclinepi.2019.02.004

38. Hart GR, Roffman DA, Decker R, et al. A multi-parameterized artificial neural network for lung cancer risk prediction. *PLoS One* 2018;13(10):e0205264. doi: 10.1371/journal.pone.0205264

39. Hornbrook MC, Goshen R, Choman E, et al. Early Colorectal Cancer Detected by Machine Learning Model Using Gender, Age, and Complete Blood Count Data. *Dig Dis Sci* 2017;62(10):2719-27. doi: 10.1007/s10620-017-4722-8

40. Miotto R, Li L, Kidd BA, et al. Deep Patient: An Unsupervised Representation to Predict the Future of Patients from the Electronic Health Records. *Sci Rep* 2016;6:26094. doi: 10.1038/srep26094

41. Richter AN, Khoshgoftaar TM. Efficient learning from big data for cancer risk modeling: A case study with melanoma. *Comput Biol Med* 2019;110:29-39. doi: 10.1016/j.compbiomed.2019.04.039

42. Roffman D, Hart G, Girardi M, et al. Predicting non-melanoma skin cancer via a multi-parameterized artificial neural network. *Sci Rep* 2018;8(1):1701. doi: 10.1038/s41598-018-19907-9

43. Schneider J, Layefsky E, Udaltsova N, et al. Validation of an Algorithm to Identify Patients at Risk for Colorectal Cancer Based on Laboratory Test and Demographic Data in Diverse, Community-Based Population. *Clin Gastroenterol Hepatol* 2020 doi: 10.1016/j.cgh.2020.04.054

44. Stark GF, Hart GR, Nartowt BJ, et al. Predicting breast cancer risk using personal health data and machine learning models. *PLoS One* 2019;14(12):e0226765. doi: 10.1371/journal.pone.0226765

45. Wang HH, Wang YH, Liang CW, et al. Assessment of Deep Learning Using Nonimaging Information and Sequential Medical Records to Develop a Prediction Model for Nonmelanoma Skin Cancer. *JAMA Dermatol* 2019 doi: 10.1001/jamadermatol.2019.2335

46. Wang YH, Nguyen PA, Islam MM, et al. Development of Deep Learning Algorithm for Detection of Colorectal Cancer in EHR Data. *Stud Health Technol Inform* 2019;264:438-41. doi: 10.3233/SHTI190259

For peer review only


For peer review only - http://bmjopen.bmj.com/site/about/guidelines.xhtml

47. Zhao D, Weng C. Combining PubMed knowledge and EHR data to develop a weighted bayesian network for pancreatic cancer prediction. *J Biomed Inform* 2011;44(5):859-68. doi: 10.1016/j.jbi.2011.05.004

48. Kinar Y, Kalkstein N, Akiva P, et al. Development and validation of a predictive model for detection of colorectal cancer in primary care by analysis of complete blood counts: a binational retrospective study. *J Am Med Inform Assoc* 2016;23(5):879-90. doi: 10.1093/jamia/ocv195

49. Gail MH, Brinton LA, Byar DP, et al. Projecting individualized probabilities of developing breast cancer for white females who are being examined annually. *J Natl Cancer Inst* 1989;81(24):1879-86. doi: 10.1093/jnci/81.24.1879

50. Lorena AC, Jacintho LF, Siqueira MF, et al. Comparing machine learning classifiers in potential distribution modelling. *Expert Systems with Applications* 2011;38(5):5268-75.

51. Usher-Smith JA, Walter FM, Emery JD, et al. Risk Prediction Models for Colorectal Cancer: A Systematic Review. *Cancer Prev Res (Phila)* 2016;9(1):13-26. doi: 10.1158/1940-6207.CAPR-15-0274

52. Gray EP, Teare MD, Stevens J, et al. Risk Prediction Models for Lung Cancer: A Systematic Review. *Clin Lung Cancer* 2016;17(2):95-106. doi: 10.1016/j.cllc.2015.11.007

53. Usher-Smith JA, Emery J, Kassianos AP, et al. Risk prediction models for melanoma: a systematic review. *Cancer Epidemiol Biomarkers Prev* 2014;23(8):1450-63. doi: 10.1158/1055-9965.EPI-14-0295

54. Rastogi T, Hildesheim A, Sinha R. Opportunities for cancer epidemiology in developing countries. *Nat Rev Cancer* 2004;4(11):909-17. doi: 10.1038/nrc1475

55. van der Ploeg T, Austin PC, Steyerberg EW. Modern modelling techniques are data hungry: a simulation study for predicting dichotomous endpoints. *BMC Med Res Methodol* 2014;14:137. doi: 10.1186/1471-2288-14-137

56. Riley RD, Ensor J, Snell KIE, et al. Calculating the sample size required for developing a clinical prediction model. *BMJ* 2020;368:m441. doi: 10.1136/bmj.m441

57. A study of cross-validation and bootstrap for accuracy estimation and model selection. Ijcai; 1995. Montreal, Canada.

For peer review only


For peer review only - http://bmjopen.bmj.com/site/about/guidelines.xhtml

58. Bergquist SL, Brooks GA, Keating NL, et al. Classifying Lung Cancer Severity with Ensemble Machine Learning in Health Care Claims Data. *Proc Mach Learn Res* 2017;68:25-38.

59. Steyerberg EW, Vickers AJ, Cook NR, et al. Assessing the performance of prediction models: a framework for traditional and novel measures. *Epidemiology* 2010;21(1):128-38. doi: 10.1097/EDE.0b013e3181c30fb2

60. Van Calster B, McLernon DJ, van Smeden M, et al. Calibration: the Achilles heel of predictive analytics. *BMC Med* 2019;17(1):230. doi: 10.1186/s12916-019-1466-7

61. Downing M. Barts Health using AI to prioritise care for colon cancer patients 2020 [Available from: https://www.bartshealth.nhs.uk/news/barts-health-using-ai-to-prioritise-care-for-high-risk-colon-cancer-patients-8867 accessed 2/11/2020].

For peer review only

**FIGURE LEGEND**

**Figure 1.** PRISMA flowchart depicting the search strategy.

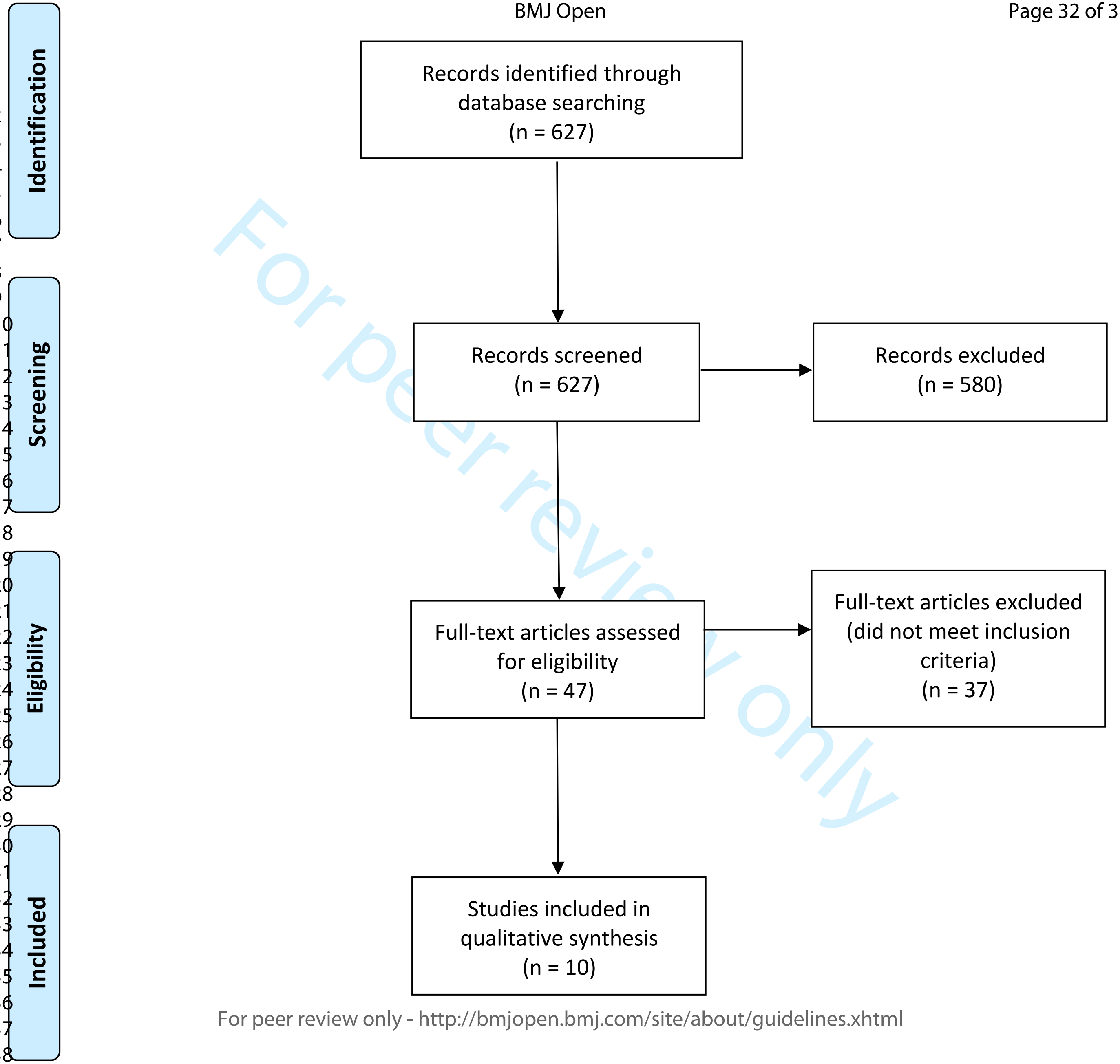


For peer review only

# Preferred Reporting Items for Systematic reviews and Meta-Analyses extension for Scoping Reviews (PRISMA-ScR) Checklist

| SECTION | ITEM | PRISMA-ScR CHECKLIST ITEM | REPORTED ON PAGE # |
|---|---|---|---|
| **TITLE** | | | |
| Title | 1 | Identify the report as a scoping review. | 1 |
| **ABSTRACT** | | | |
| Structured summary | 2 | Provide a structured summary that includes (as applicable): background, objectives, eligibility criteria, sources of evidence, charting methods, results, and conclusions that relate to the review questions and objectives. | 2 and 3 |
| **INTRODUCTION** | | | |
| Rationale | 3 | Describe the rationale for the review in the context of what is already known. Explain why the review questions/objectives lend themselves to a scoping review approach. | 7 |
| Objectives | 4 | Provide an explicit statement of the questions and objectives being addressed with reference to their key elements (e.g., population or participants, concepts, and context) or other relevant key elements used to conceptualize the review questions and/or objectives. | 7 |
| **METHODS** | | | |
| Protocol and registration | 5 | Indicate whether a review protocol exists; state if and where it can be accessed (e.g., a Web address); and if available, provide registration information, including the registration number. | NA |
| Eligibility criteria | 6 | Specify characteristics of the sources of evidence used as eligibility criteria (e.g., years considered, language, and publication status), and provide a rationale. | 9 |
| Information sources* | 7 | Describe all information sources in the search (e.g., databases with dates of coverage and contact with authors to identify additional sources), as well as the date the most recent search was executed. | 9 |
| Search | 8 | Present the full electronic search strategy for at least 1 database, including any limits used, such that it could be repeated. | 9 |
| Selection of sources of evidence† | 9 | State the process for selecting sources of evidence (i.e., screening and eligibility) included in the scoping review. | 9 |
| Data charting process‡ | 10 | Describe the methods of charting data from the included sources of evidence (e.g., calibrated forms or forms that have been tested by the team before their use, and whether data charting was done independently or in duplicate) and any processes for obtaining and confirming data from investigators. | 13, 14, 15, and 16 |
| Data items | 11 | List and define all variables for which data were sought and any assumptions and simplifications made. | 13, 14, 15, and 16 |
| Critical appraisal of individual sources of evidence§ | 12 | If done, provide a rationale for conducting a critical appraisal of included sources of evidence; describe the methods used and how this information was used in any data synthesis (if appropriate). | NA |

St. Michael's
Inspired Care. Inspiring Science.



For peer review only - http://bmjopen.bmj.com/site/about/guidelines.xhtml

| SECTION | ITEM | PRISMA-ScR CHECKLIST ITEM | REPORTED ON PAGE # |
|---|---|---|---|
| Synthesis of results | 13 | Describe the methods of handling and summarizing the data that were charted. | 9 and 10 |
| **RESULTS** | | | |
| Selection of sources of evidence | 14 | Give numbers of sources of evidence screened, assessed for eligibility, and included in the review, with reasons for exclusions at each stage, ideally using a flow diagram. | 11 |
| Characteristics of sources of evidence | 15 | For each source of evidence, present characteristics for which data were charted and provide the citations. | 13, 14, 15, and 16 |
| Critical appraisal within sources of evidence | 16 | If done, present data on critical appraisal of included sources of evidence (see item 12). | NA |
| Results of individual sources of evidence | 17 | For each included source of evidence, present the relevant data that were charted that relate to the review questions and objectives. | 13, 14, 15, and 16 |
| Synthesis of results | 18 | Summarize and/or present the charting results as they relate to the review questions and objectives. | 11,12, 17, and 18 |
| **DISCUSSION** | | | |
| Summary of evidence | 19 | Summarize the main results (including an overview of concepts, themes, and types of evidence available), link to the review questions and objectives, and consider the relevance to key groups. | 18, 19, 20, 21, and 22 |
| Limitations | 20 | Discuss the limitations of the scoping review process. | 22 and 23 |
| Conclusions | 21 | Provide a general interpretation of the results with respect to the review questions and objectives, as well as potential implications and/or next steps. | 23 |
| **FUNDING** | | | |
| Funding | 22 | Describe sources of funding for the included sources of evidence, as well as sources of funding for the scoping review. Describe the role of the funders of the scoping review. | 23 and 24 |

JBI = Joanna Briggs Institute; PRISMA-ScR = Preferred Reporting Items for Systematic reviews and Meta-Analyses extension for Scoping Reviews.
* Where *sources of evidence* (see second footnote) are compiled from, such as bibliographic databases, social media platforms, and Web sites.
† A more inclusive/heterogeneous term used to account for the different types of evidence or data sources (e.g., quantitative and/or qualitative research, expert opinion, and policy documents) that may be eligible in a scoping review as opposed to only studies. This is not to be confused with *information sources* (see first footnote).
‡ The frameworks by Arksey and O'Malley (6) and Levac and colleagues (7) and the JBI guidance (4, 5) refer to the process of data extraction in a scoping review as data charting.
§ The process of systematically examining research evidence to assess its validity, results, and relevance before using it to inform a decision. This term is used for items 12 and 19 instead of "risk of bias" (which is more applicable to systematic reviews of interventions) to include and acknowledge the various sources of evidence that may be used in a scoping review (e.g., quantitative and/or qualitative research, expert opinion, and policy document).

*From:* Tricco AC, Lillie E, Zarin W, O'Brien KK, Colquhoun H, Levac D, et al. PRISMA Extension for Scoping Reviews (PRISMAScR): Checklist and Explanation. Ann Intern Med. 2018;169:467–473. doi: 10.7326/M18-0850.


For peer review only - http://bmjopen.bmj.com/site/about/guidelines.xhtml
